# Synthesis, Characterization, and *in vivo* Evaluation of a Novel Potent Autotaxin-Inhibitor

**DOI:** 10.3389/fphar.2021.699535

**Published:** 2022-01-18

**Authors:** Daniel Hunziker, Sabrina Reinehr, Marina Palmhof, Natalie Wagner, Thomas Biniasch, Gesa Stute, Patrizio Mattei, Petra Schmitz, Patrick DiGiorgio, Jérôme Hert, Markus G. Rudolph, Joerg Benz, Martine Stihle, Bernard Gsell, Stephan Müller, Rodolfo Gasser, Nina Schonhoven, Christoph Ullmer, Stephanie C. Joachim

**Affiliations:** ^1^ F. Hoffmann-La Roche Ltd., Pharma Research and Early Development, Therapeutic Modalities, Small Molecule Research, Roche Innovation Center Basel, Basel, Switzerland; ^2^ Experimental Eye Research Institute, University Eye Hospital, Ruhr-University Bochum, Bochum, Germany; ^3^ F. Hoffmann-La Roche Ltd., Pharma Research and Early Development, Pharmaceutical Sciences, Roche Innovation Center Basel, Basel, Switzerland; ^4^ F. Hoffmann-La Roche Ltd., Pharma Research and Early Development, Ophthalmology Discovery, Roche Innovation Center Basel, Basel, Switzerland

**Keywords:** autoimmune glaucoma model, ischemia, glaucoma, autotaxin (ATX), lysophosphatidic acid, optic nerve, retina, retinal ganglion ceils

## Abstract

The autotaxin-lysophosphatidic acid (ATX-LPA) signaling pathway plays a role in a variety of autoimmune diseases, such as rheumatoid arthritis or neurodegeneration. A link to the pathogenesis of glaucoma is suggested by an overactive ATX-LPA axis in aqueous humor samples of glaucoma patients. Analysis of such samples suggests that the ATX-LPA axis contributes to the fibrogenic activity and resistance to aqueous humor outflow through the trabecular meshwork. In order to inhibit or modulate this pathway, we developed a new series of ATX-inhibitors containing novel bicyclic and spirocyclic structural motifs. A potent lead compound (IC_50_ against ATX: 6 nM) with good *in vivo* PK, favorable *in vitro* property, and safety profile was generated. This compound leads to lowered LPA levels *in vivo* after oral administration. Hence, it was suitable for chronic oral treatment in two rodent models of glaucoma, the experimental autoimmune glaucoma (EAG) and the ischemia/reperfusion models. In the EAG model, rats were immunized with an optic nerve antigen homogenate, while controls received sodium chloride. Retinal ischemia/reperfusion (I/R) was induced by elevating the intraocular pressure (IOP) in one eye to 140 mmHg for 60 min, followed by reperfusion, while the other untreated eye served as control. Retinae and optic nerves were evaluated 28 days after EAG or 7 and 14 days after I/R induction. Oral treatment with the optimized ATX-inhibitor lead to reduced retinal ganglion cell (RGC) loss in both glaucoma models. In the optic nerve, the protective effect of ATX inhibition was less effective compared to the retina and only a trend to a weakened neurofilament distortion was detectable. Taken together, these results provide evidence that the dysregulation of the ATX-LPA axis in the aqueous humor of glaucoma patients, in addition to the postulated outflow impairment, might also contribute to RGC loss. The observation that ATX-inhibitor treatment in both glaucoma models did not result in significant IOP increases or decreases after oral treatment indicates that protection from RGC loss due to inhibition of the ATX-LPA axis is independent of an IOP lowering effect.

## 1 Introduction

Autotaxin (ATX) or ectonucleotide pyrophosphatase/phosphodiesterase 2 (ENPP2) is an extracellular zinc-dependent enzyme that converts lysophosphatidyl choline (LPC) into the bioactive phospholipid lysophosphatidic acid (LPA) and choline. LPA acts as a bioactive growth factor-like phospholipid consisting of a glycerol backbone, a phosphate group and a fatty acid chain that can vary in length and degree of saturation. LPA mediates most of its biological effects through six G-protein-coupled receptors (GPCRs) known as LPAR1-6, thus stimulating a variety of biological functions including proliferation, migration, invasion, and survival of many cell types. ATX has attracted substantial interest over the last decade as a potential therapeutic target since the ATX-LPA axis was shown to play a key role in a variety of physiological and pathological conditions including fibrotic diseases, arthritis, glaucoma, and a number of other disorders (Matralis et al., 2019). The first and only ATX-inhibitor to enter clinical trials, Ziritaxestat (GLPG-1690), for idiopathic pulmonary fibrosis has advanced to clinical phase 3 (Maher et al., 2018). The ISABELA phase 3 trial, however, was discontinued recently as the benefit-risk profile no longer supported continuing these studies.

**FIGURE 1:**
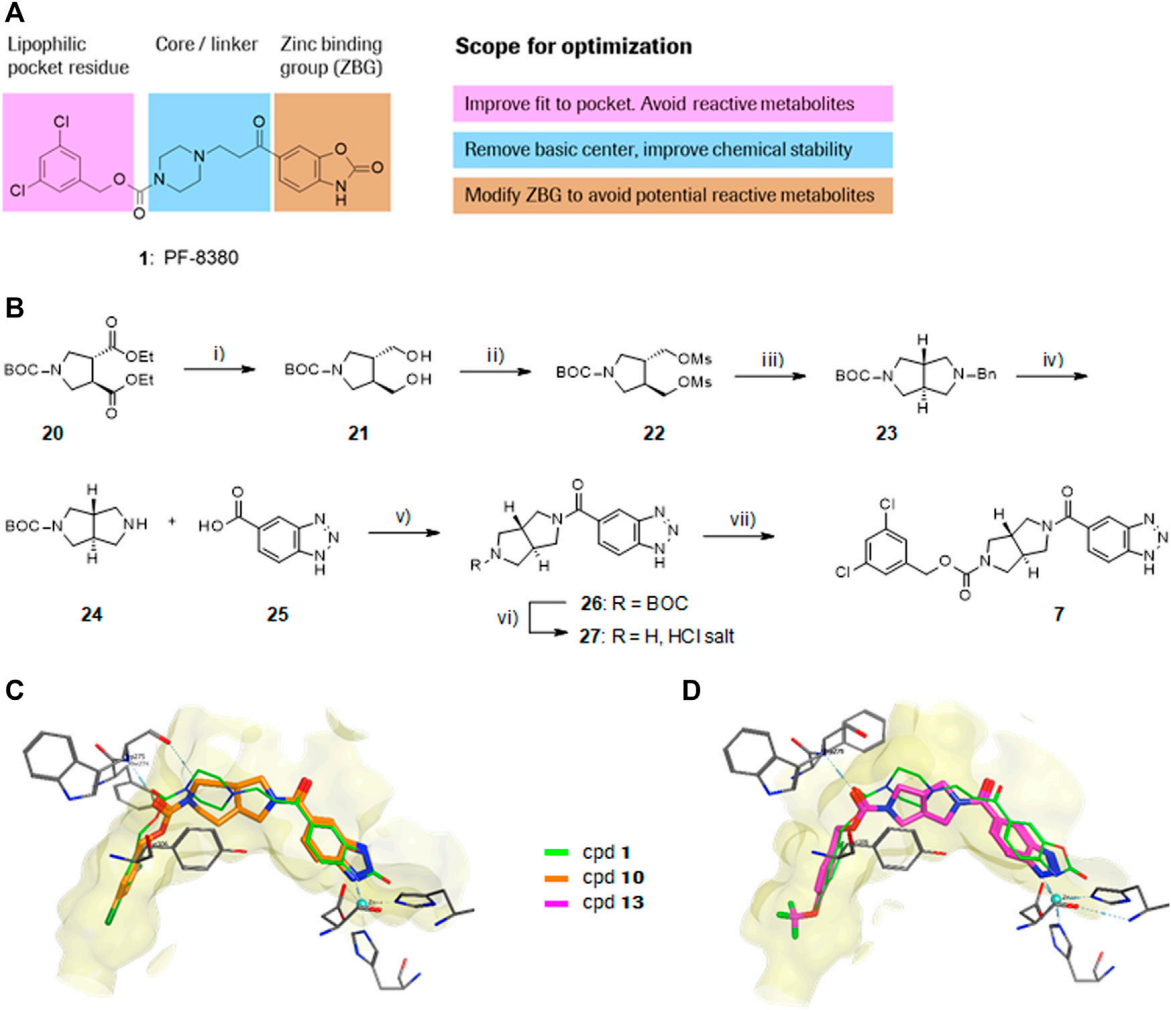
Structure, synthesis and co-crystal structures of ATX-inhibitors. **(A)** Structure of PF-8380 (**1**) with an outline of the general approach for modification and optimization. **(B)** Synthesis of ATX-inhibitor **7**. i) 4M LiBH_4_, THF, RT, 91–95% crude; ii) MsCl, NEt_3_, DCM, -10°C, 94% crude; iii) BnNH_2_, K_2_CO_3_, CH_3_CN, 95°C, 24 h, 53%; iv) H_2_, Pd/C, 1 bar, MeOH, 96%; v) HATU, NMM, DMF, RT, 16 h, 83%; vi) HCl/^i^PrOH (∼5–6M), RT, 87%; vii) (3,5-dichlorophenyl)methanol, CDI, NEt_3_, CH_3_CN, reflux, 87%. **(C)** X-ray crystal structures of human ATX in complex with **1** and **10** (overlay). Both inhibitors interact with the zinc ion in the active site (light blue) and stretch into a lipophilic pocket which corresponds to the fatty acid binding site of LPC (left). Besides the fit to the lipophilic pocket, other key interactions for **10** are the binding of the benzotriazole to the active site zinc ion and an H-bond of the carbamate oxygen to the backbone NH of Trp275. **(D)** X-ray crystal structures of **1** and **13** (overlay), with an essentially identical pattern of contacts with the enzyme.

PF-8380 (**1**) was one of the early ATX-inhibitor tool compounds published by Pfizer, which showed inhibition of LPA generation *in vivo* (Gierse et al., 2010). In order to obtain a new set of proprietary ATX-inhibitors, we initially used **1** as a template for structure-based inhibitor optimization with subsequent removal of liabilities identified for **1**. Basically, **1** can be divided into three parts, which can to some extent be optimized individually (Figure 1A) while keeping the fit of the molecule to the active site of ATX preserved. One important liability for **1** was substantial formation of reactive metabolites as observed by glutathione (GSH) addition to both the benzoxazol-2-one zinc-binding group (ZBG) and to the dichlorophenyl moiety. In addition, the 1,4-arrangement of a carbonyl group and charged nitrogen of the piperazine core could potentially result in some chemical instability. Besides this, metabolic stability of **1** was found to be limited and solubility was determined to be very low (Table 1). With this approach, we pursued a similar path as described by others for another set of ATX-inhibitors (Kuttruff et al., 2017).

**TABLE 1:** Initial screening of central cores and zinc binding groups.

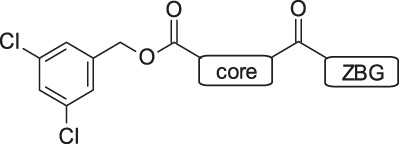	
Cmpd	Core	ZBG	hATX IC_50_ [nM]	LogD (pH 7.4)	Solubility [µg/mL], pH 6.5	Mic CL h/m/r [µL/m/mg prot]	GSH adducts
**1**	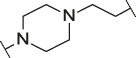	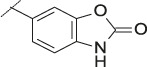	3	2.7	<1	65/78/84	pos
**2**	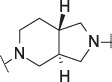	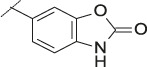	3	3.7	<1	39/74/*nd*	pos
**3**	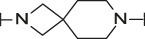	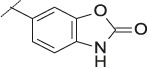	220	3.6	<1	92/81/*nd*	*nd*
**4**	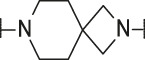	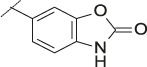	17	3.6	<1	54/149/*nd*	pos
**5**	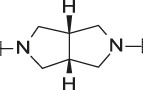	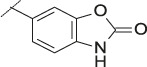	65	3.3	19	64/288/335	pos
**6**	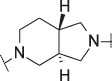	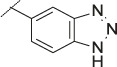	4	3.5	9	23/65/*nd*	pos
**7**	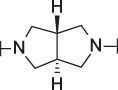	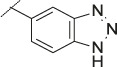	4	3.1	9	16/17/10	pos
**8**	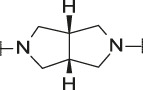	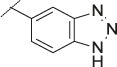	265	3.1	34	23/68/*nd*	pos
**9**	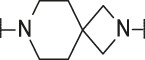	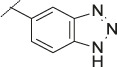	67	3.6	3	30/63/29	pos
**10**	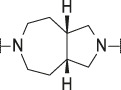	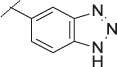	1	3.5^a^	7	252/727/*nd*	*nd*

a Calculated value; *nd* = not determined. Initial screening of central cores and zinc binding groups (ZBG).

Initial expression analysis of ATX revealed highest expression in adipocytes and retina (Giganti et al., 2008). Cellular resolution of ATX expression in the eye, analyzed by *in situ* hybridization, is restricted to epithelial cells of the ciliary body and retinal pigment epithelial cells (Narita et al., 1994). Both are secretory epithelial cells that have anatomically tight junctions and are localized on stroma, which are rich in capillaries with fenestrated endothelium and might be the main producer of abundant ATX found in human vitreous fluid (Abu El-Asrar et al., 2013) and aqueous humor (Iyer et al., 2012). The link to the pathogenesis of primary open-angle glaucoma (POAG) is suggested by elevated levels of ATX, LPA, and LPC in North American (in both Caucasians and African Americans) and Japanese POAG patients that correlated with elevation of intraocular pressure (IOP) (Honjo et al., 2018; Ho et al., 2020). Also, lysophosphatidic lipase D activity was elevated in the aqueous humor of POAG patients relative to age matched control (cataract) subjects (Iyer et al., 2012). A recent study suggests that aqueous ATX levels can distinguish glaucoma subtypes and serve as a promising biomarker for open-angle glaucoma subtypes (Igarashi et al., 2021).

Trabecular meshwork has been proposed as the relevant target tissue where ATX was induced during mechanical stress in isolated human cells (Iyer et al., 2012). The resulting LPA regulates plasticity and fibrogenic activity in the Schlemm’s canal (Pattabiraman et al., 2014). The product of ATX, LPA, has in turn been demonstrated to impair aqueous humor outflow facility in the porcine enucleated eye (Mettu et al., 2004) and increase IOP of rabbit eyes after intracameral instillation (Nakamura et al., 2021). Inhibition of lysophosphatidic lipase D activity by topical and intracameral delivery of a chemical ATX-inhibitor significantly decreases IOP in rabbits, suggesting that ATX is a potential therapeutic target for lowering IOP in glaucoma patients (Iyer et al., 2012).

The role of ATX in POAG patients seems to go beyond IOP control. ATX expression was increased by more than 10-fold in astrocytes isolated from the optic nerve head of glaucoma patients (Hernandez et al., 2002). It was already shown that stearoyl-LPA or hypoxia reduced the viability of retinal ganglion cells (RGCs) and a LPA1R antagonist was neuroprotective and led to RGC survival in a mouse model of oxygen-induced retinopathy (Yang et al., 2009). As glaucomatous optic neuropathy involves specific degeneration of the RGCs, an overactive ATX-LPA axis in glaucoma might also contribute to neuropathy. We sought to elaborate protective effects by ATX inhibition using lead compound **13** (ATX-i) in two glaucoma models of RGC degeneration, an ischemia/reperfusion (I/R) model and an experimental autoimmune glaucoma (EAG) model.

In the EAG model, neuroprotective mechanisms can be analyzed independently from an elevated IOP. Here, an immunization with antigens, such as the optic nerve antigen homogenate (ONA) leads to a degeneration of RGCs and optic nerve axons after 28 days (Laspas et al., 2011; Noristani et al., 2016). In previous studies, an increase in microglia numbers as well as an activation of the complement system were noted prior to cell death (Noristani et al., 2016; Reinehr et al., 2016). Furthermore, antibody deposits were detected in glaucomatous retinas (Joachim et al., 2012a), which might contribute to retinal and optic nerve degeneration in the EAG model.

Retinal I/R is a common rodent model to induce retinal damage. In the majority of studies, it is achieved through an IOP increase above the systolic level for a defined time period (Adachi et al., 1996; Joachim et al., 2012b). In the I/R model, our group and others described degeneration of RGCs, a thinning of the inner retinal layers, and consequently observed impaired retinal function by decreased a-wave and b-wave electroretinogram (ERG) amplitudes in the ischemic eyes (Schmid et al., 2014; Wu et al., 2020). In a recent study, we noted that ischemic processes have a strong destructive effect on RGCs. Less RGCs could be detected on protein level already 2 h after I/R and on mRNA level at 12 h (Palmhof et al., 2019). In addition, 2 h after I/R induction, microglia/macrophages are already in an active state (Wagner et al., 2020).

The goal of this project was to investigate if an orally bioavailable ATX-inhibitor protects from damage of the retina in two different preclinical glaucoma models. For this purpose, a novel ATX-inhibitor (**13**) from a new chemical series amenable for chronic oral delivery was tested in the EAG as well as in an I/R model. We investigated if retinal function and structure could be protected through the ATX-inhibitor in both models.

## 2 Materials and Methods

### 2.1 Synthesis of ATX-Inhibitors

A representative synthesis for an inhibitor with a new bicyclic central core is given in this section for compound **7**. Detailed syntheses and analytical data for the other compounds **2**–**6** and **8**–**15** described in this paper are provided in the Supplementary Material Section 1.1, S1.1–S1.13. Synthesis of compound **7** was done according to Figure 1B:

(*3R,4R*)-tert-Butyl 3,4-bis(hydroxymethyl)pyrrolidine-1-carboxylate (**21**).

Lithium borohydride (3.60 g, 165 mmol) was added at −5°C in several portions to a solution of (*3R,4R*)-1-tert-butyl 3,4-diethyl pyrrolidine-1,3,4-tricarboxylate (20.9 g, 66.1 mmol) in tetrahydrofuran (350 ml). After 2 h, the ice bath was removed, then after 18 h, the reaction was stopped by dropwise addition of methanol (150 ml), while keeping the reaction temperature at 25°C under ice cooling. After 2 h, the hydrogen release had stopped and the mixture was evaporated (Rodríguez Sarmiento et al., 2003). The residue was partitioned between ethyl acetate and 50% aq. ammonium chloride solution. The organic layer was washed with brine, dried over magnesium sulfate, filtered, and evaporated to afford the title compound **21** (14.3 g, 94%) as a colorless oil, MS: 254.5 [M + Na]^+^. ^1^H NMR (CDCl_3_, 300 MHz) δ 3.68 (br s, 2H), 3.4–3.6 (m, 4H), 3.29 (br s, 2H), 2.93 (br t, 2H, J = 9.5 Hz), 2.13 (br s, 2H), 1.39 (s, 9H). (*3R,4R*)-tert-Butyl 3,4-bis((methylsulfonyloxy)methyl)pyrrolidine-1-carboxylate (**22**).

A solution of methanesulfonyl chloride (21.1 g, 185 mmol) in dichloromethane (30 ml) was added dropwise at 0°C to a solution of (*3R,4R*)-tert-butyl 3,4-bis(hydroxymethyl)pyrrolidine-1-carboxylate (**21**; 14.23 g, 61.5 mmol) and *N,N*-diisopropylethylamine (47.7 g, 369 mmol) in dichloromethane (300 ml), then after 90 min, the reaction mixture was washed with water. The organic layer was washed with 50% aq. sodium hydrogencarbonate solution, water and brine, dried over magnesium sulfate, and evaporated to afford the title compound **22** (22.4 g, 94%) as a dark red oil, MS: 332.4 [M–isobutene + H]^+^.

^1^H NMR (CDCl_3_, 300 MHz) δ 4.26 (br d, 4H, J = 4.8 Hz), 3.6–3.7 (m, 2H), 3.25 (br dd, 2H, J = 7.0, 11.2 Hz), 3.06 (s, 6H), 2.53 (br s, 2H), 1.46 (m, 9H).

(*3aS,6aS*)-tert-Butyl 5-benzylhexahydropyrrolo [3,4-c]pyrrole-2(*1H*)-carboxylate (**23**).

To a clear orange solution of (*3R,4R*)-tert-butyl 3,4-bis((methylsulfonyloxy)methyl)-pyrrolidine-1-carboxylate (**22**; 13.42 g, 34.6 mmol) in acetonitrile (200 ml) were added potassium carbonate (23.9 g, 173 mmol) and phenylmethanamine (11.1 g, 104 mmol). The suspension was heated to 95°C for 24 h, then after cooling, the reaction mixture was partitioned between ethyl acetate 10% and aq. citric acid solution. The aqueous layer was basified to pH 7 with sodium hydrogen carbonate solution and extracted 4 times with ethyl acetate. The combined ethyl acetate layers were dried over magnesium sulfate, filtered, and evaporated to afford the title compound **23** (5.52 g, 53%) as light yellow solid, MS: 303.6 [M + H]^+^.

^1^H NMR (CDCl_3_, 300 MHz) δ 7.2–7.3 (m, 5H), 3.8–3.9 (m, 2H), 3.4–3.7 (m, 2H), 2.8–3.0 (m, 4H), 2.59 (dt, 2H, J = 3.5, 9.6 Hz), 2.1–2.3 (m, 2H), 1.45 (s, 9H).

(*3aS,6aS*)-tert-Butyl hexahydropyrrolo [3,4-c]pyrrole-2(*1H*)-carboxylate (**24**).

To a solution of (*3aS,6aS*)-tert-butyl 5-benzylhexahydropyrrolo [3,4-c]pyrrole-2(*1H*)-carboxylate (**23**; 2.22 g, 7.34 mmol) in methanol (20 ml) was added palladium (10% on carbon, 220 mg, 7.34 mmol) and the reaction mixture was stirred under a hydrogen atmosphere (1 bar) at room temperature for 24 h, then insoluble material was removed by filtration through diatomaceous earth. The filtrate was concentrated to produce the title compound **24** (1.60 g, 100%). White waxy solid, MS: 213.5 (M + H)^+^.

^1^H NMR (CDCl_3_, 300 MHz) δ 3.5–3.7 (m, 2H), 2.9–3.1 (m, 4H), 2.63 (dt, 2H, J = 4.0, 9.9 Hz), 2.0–2.3 (m, 2H), 1.87 (br s, 1H), 1.46 (s, 9H).

(*3aS,6aS*)-tert-Butyl 5-(*1H*-benzo [d][1,2,3]triazole-5-carbonyl)hexahydropyrrolo [3,4-c]pyrrole-2(*1H*)-carboxylate (**26**).

*O*-(7-azabenzotriazol-1-yl)-*N,N,N′,N′*-tetramethyluronium hexafluoro-phosphate (HATU, 915 mg, 2.41 mmol) was added at 0°C to a solution of (*3aS,6aS*)-tert-butyl hexahydropyrrolo [3,4-c]pyrrole-2(*1H*)-carboxylate (**24**; 511 mg, 2.41 mmol), *N*-methylmorpholine (730 mg, 7.22 mmol) and *1H*-benzo [d][1,2,3]triazole-5-carboxylic acid (**25**, 393 mg, 2.41 mmol) in *N,N*-dimethylformamide (13 ml). The reaction mixture was allowed to reach room temperature over 16 h and was then partitioned between ethyl acetate and sat aq. sodium hydrogen carbonate solution. The organic layer was washed with water and brine dried over magnesium sulfate filtered and evaporated. Chromatography (silica gel; gradient dichloromethane to dichloromethane/methanol/25% aq. Ammonia solution 90:10:0.25) afforded the title compound **26** (718 mg, 83%) as a light yellow foam, MS: 358.5 [M + H]^+^.

^1^H NMR (CDCl_3_, 300 MHz) δ 14.43 (br s, 1H), 8.03 (br s, 1H), 7.82 (br d, 1H, J = 6.9 Hz), 7.59 (br d, 1H, J = 9.1 Hz), 3.9–4.1 (m, 1H), 3.5–3.8 (m, 3H), 3.3–3.5 (m, 2H), 3.0–3.2 (m, 2H), 2.2–2.5 (m, 2H), 1.49 (s, 9H).

(*1H*-Benzo [d][1,2,3]triazol-5-yl) ((*3aR,6aR*)-hexahydropyrrolo [3,4-c]pyrrol-2(*1H*)-yl)methanone hydrochloride (**27**).

To a light yellow solution of (*3aS,6aS*)-tert-butyl 5-(*1H*-benzo [d][1,2,3]triazole-5-carbonyl)hexahydropyrrolo [3,4-c]pyrrole-2(*1H*)-carboxylate (**26**, 733 mg, 2.05 mmol) in 2-propanol (8 ml) was added hydrochloric acid solution (5–6 M in 2-propanol, 9 ml, 45 mmol) at room temperature and then the reaction mixture was evaporated. The residue was triturated with ethyl acetate and the precipitate was collected by filtration to afford the title compound **27** (523 mg, 87%). Light yellow solid, MS: 256.5 [M–H]^–^.

^1^H NMR ((CD_3_)_2_SO, 300 MHz) δ 9.68 (br s, 2H), 8.11 (s, 1H), 7.95 (br d, 1H, J = 8.5 Hz), 7.59 (d, 1H, J = 8.5 Hz), 3.77 (dd, 1H, J = 6.5, 10.9 Hz), 3.3–3.6 (m, 5H), 2.8–3.0 (m, 3H), 2.2–2.4 (m, 2H).

(*3aS,6aS*)-5-(*1H*-Benzotriazole-5-carbonyl)-hexahydro-pyrrolo [3,4-c]pyrrole-2-carboxylic acid 3,5-dichloro-benzyl ester (**7**).

To a solution of (3,5-dichlorophenyl)methanol (21.4 mg, 121 µmol) in acetonitrile (5 ml) was added *N,N′*-carbonyldiimidazole (20.6 mg, 127 µmol) at room temperature, then after 3 h, triethylamine (61.3 mg, 606 µmol) and (*1H*-benzo [d][1,2,3]triazol-5-yl) ((*3aR,6aR*)-hexahydropyrrolo [3,4-c]pyrrol-2(*1H*)-yl)methanone hydrochloride (**27**; 40 mg, 121 µmol) were added and the reaction mixture was heated at reflux. After 16 h, the reaction mixture was partitioned between ethyl acetate and sat. aq. ammonium chloride solution, the organic layer was washed with sat. aq. sodium hydrogen carbonate solution and brine, dried over magnesium sulfate, filtered, and evaporated. Chromatography (silica gel; gradient dichloromethane to dichloromethane/methanol/25% aq. ammonia solution 90:10:0.25) produced the title compound **7** (38 mg, 68%) as a light-yellow foam. ^1^H NMR (CDCl_3_, 300 MHz) δ 14.60 (br s, 1H), 8.00 (br s, 1H), 7.80 (br s, 1H), 7.58 (br d, 1H, J = 8.7 Hz), 7.2–7.4 (m, 3H), 5.09 (d, 2H, J = 3.0 Hz), 4.0–4.1 (m, 1H), 3.8–3.9 (m, 1H), 3.6–3.8 (m, 2H), 3.3–3.5 (m, 2H), 3.1–3.3 (m, 2H), 2.2–2.5 (m, 2H). LC-HRMS (m/z) [M + H]^+^ calcd for [C_21_H_19_Cl_2_N_5_O_3_+H]^+^: 460.0938, found: 460.0943, UV purity (230–300 nm): 99%.

### 2.2 *In vitro* Studies

#### 2.2.1 Autotaxin Inhibition Assay

Inhibition of human ATX was determined using the fluorogenic Amplex-Red *in vitro* assay, which was adapted from Ferry et al. (Ferry et al., 2008). DMSO-containing compound solutions were transferred to a 384-well assay plate (Greiner #781096, Monroe, NC, United States) and 6.6 nM in-house generated ATX enzyme in assay buffer (50 mM Tris-HCl, 120 mM NaCl, 20 mM CaCl_2_, 5 mM KCl, 0.01% Triton-X100, pH 8.0) was added. After a 10-min incubation at room temperature, choline oxidase (7.3 U/ml; Sigma-Aldrich #C5896-1KU, St. Louis, MO, United States) and horseradish peroxidase (14.7 U/ml; F. Hoffmann-La Roche Ltd. #11378783, 1MU/4.311 g lyophilizate, Basel, Switzerland) were added and mixed. Thereafter, 110 μM LPC 18:1 (Avanti Polar Lipids #845875SP, Alabaster, AL, United States) and 183.3 μM Amplex Red (Chemodex #A-022, St. Gallen, Switzerland) were added. Plates were sealed and incubated for 5 min. The final assay concentrations were ATX 3 nM, choline oxidase 2 U/ml, horseradish peroxidase 4 U/ml, LPC 18:1 30 μM, Amplex Red 50 μM, and DMSO 2%. Background values were measured within 15 min and activity values after 90 min of incubation (Perkin Elmer Envision; Ex 535 nm/Em 587 nm; Perkin Elmer AG, Schwerzenbach, Switzerland).

#### 2.2.2 HT-Solubility Assay

This assay was performed on a TECAN station starting from 10 mM DMSO stock solutions of the test compounds. Two aliquots of the test compound were dried and dissolved in phosphate buffer at pH 6.5. Sonication supports optimal re-solubilization. The solutions were then filtered and diluted (3 different dilution levels for each compound) followed by quantification of the dissolved material with Rapid-Fire MS. Quantification was performed for each test compound with a 6-point calibration curve prepared with the same DMSO starting solution.

#### 2.2.3 Glutathione Addition Screening

All sample handling was performed based on a 96-well plate format using an automated pipetting system. The test compounds, positive controls (diclofenac, troglitazone, nefazodone, 4-[[1-(4-fluorophenyl)-2-methyl-1H-imidazol-4-yl]ethynyl]-2-methylpyridine) and solvent control (DMSO) were incubated with human liver microsomes supplemented with NADPH and reduced GSH. Stable GSH conjugates whose production can be assumed to be the result of chemically reactive metabolites were detected using a quadrupole time of flight mass spectrometer run in untargeted full scan accurate mass MSE mode (Waters Xevo G1 QTOFMSE; Waters GmbH, Eschborn, Germany). This generic GSH adduct detection method and the assay workflow are taking advantage of the specific fragmentation behavior of the peptide moiety upon collision induced dissociation of GSH adducts and so are largely independent of the conjugate (“X”) itself.

#### 2.2.4 Artificial Membrane Permeability Assay

The assay was performed in an automated fashion on 96-well microplates. The permeation of drugs was measured using a “sandwich” construction: A filter-plate was coated with phospholipids (membrane) and placed into a donor compartment containing a compound/buffer solution. Finally, the top of filter-plate was filled with buffer solution (acceptor). Donor concentration was measured at t-start (reference) and compared with the donor and acceptor concentration after a certain time (t-end) to calculate the extent of passage of the compound through the membrane. The main readout of the assay was the permeability value Pe expressed in 10^–6^ * cm/s. Secondary readouts determined were the amount of compound in the donor and acceptor compartments as well as the retention in the membrane. Depending on the permeation rate and the membrane retention the compounds were classified as low (Pe < 0.2 and membrane < 20%) or medium and high (Pe ≥ 0.2 or Pe < 0.2 and membrane ≥ 20%). Each sample was measured in triplicate and a standard deviation was determined for the permeation constant Pe.

#### 2.2.5 Lipophilicity

The experiment started with the accurate coating of the hydrophobic layer (0.45 µm PVDF membranes), which was fixed on the bottom of each DIFI©-tube (Weidmann Medical Technology AG, Rapperswil, Switzerland). The coated membranes were then connected to a 96-well plate which has been prefilled with exactly 150 µl of the selected aqueous buffer solution (25 mM phosphate buffer, pH 7.4). The buffer solution contains already the compound of interest with a maximum starting concentration of 85 µM. To expand the measurement range down to logD = −0.5 and up to logD = +4, it is necessary to carry out the procedure at two different octanol/water ratios: One with an excess of octanol for hydrophilic compounds (logD < 1) and one with a low volume of octanol for the lipophilic compounds (logD > 1). Therefore, parts of the DIFI©-tubes were filled with 15 µl 1-octanol and another part with 1 µl 1-octanol. The resulting sandwich guarantees that the membrane was completely dipped in the buffer solution. The plate was then sealed and shaken for 12 h at room temperature (23°C). During this time, the substance is distributed between the layer, the octanol, and the buffer solution. After having reached a distribution equilibrium, the DIFI©-tubes were easily disassembled from the top of the 96-well plate, so that the remaining sample concentration in the aqueous phase could be analyzed by LC/MS.

#### 2.2.6 *In vitro* Microsomal Stability Assay

Incubations of a test compound at a concentration of 1 µM with microsomes (0.5 mg/ml) from the appropriate species (human, mouse, or rat) plus cofactor NADPH were performed in 96-well plates at 37°C on a TECAN automated liquid handling system (Tecan Group Ltd., Männedorf, Switzerland). After pre-incubation of the test compound with the microsomes for 10 min, the enzymatic reaction was started by the addition of cofactors. Aliquots of the incubations are removed at 1, 3, 6, 9, 15, 25, 35, and 45 min and are then quenched with 1:3 (v/v) acetonitrile containing internal standards. Samples were then cooled and centrifuged before analysis of the supernatant by LC-MS/MS. Logarithmic Peak area ratios (test compound peak area/internal standard peak area) were plotted against incubation time and a linear fit made to the data with emphasis upon the initial rate of compound disappearance. The slope of the fit was then used to calculate the intrinsic clearance: Clint (µL/min/mg protein) = −slope (min-1) * 1,000/[protein concentration (mg/ml)].

### 2.3 *In vivo* Studies

#### 2.3.1 Animals

All procedures concerning animals adhered to the ARVO statement for the use of animals in ophthalmic and vision research. Experiments performed at F. Hoffmann-La Roche (Basel) complied with the Swiss Federal and Cantonal laws on animal research and AAALAC regulations and received prior approval by the Cantonal Veterinary Office. The animal care committee of North Rhine-Westphalia (Germany) approved all *in vivo* experiments of the EAG and I/R studies. For the EAG model, male Lewis rats (Charles River, Sulzfeld, Germany), 6 weeks of age, were used. The I/R model was performed on 7- to 8-weeks old Brown-Norway rats (Charles River). All animals were kept under environmentally controlled conditions with free access to chow and water. Detailed observations and health checks, including eye exams, were performed regularly.

#### 2.3.2 Single Dose PK and Exposure Analysis From *in vivo* Studies

Exposures in animals treated with ATX-inhibitors were determined from K_2_EDTA treated plasma by LC-MS/MS at different time points. 25 µL of plasma was used for a single concentration determination. Concentration/time profiles were analyzed with MS Excel (Microsoft Corporation, Redmond, WA, United States).

For PK experiments for **7**, **13**, **14**, and **15**, respectively, animals were treated either iv or po and exposures were determined at different time points after compound administration. Suitable formulations for **13** were, for example, NMP:Tris buffer pH 8.5 (30%/70%) for iv administration with a nominal drug concentration of 1 mg/ml (dose 2 mg/kg) and a suspension in NMP:gelatine/NaCl (10%/90%) for po administration with a nominal drug concentration of 2.5 mg/ml (dose 10 mg/kg). PK parameters in Table 2 were estimated from the concentration time profiles using Phoenix WinNonlin software (Certara, Princeton, NJ, United States).

**TABLE 2:** Pharmacokinetic data in rats. **(A)** Intravenous bolus administration for compound **7**, **13**, **14**, and **15**. **(B)** Oral administration for compound **7**, **13**, **14**, **15** by gavage and food admix for compound **13.** Abbreviations: AUC: area under the curve; CLp: plasma clearance; n: number of animals; FA: food admix; Oral F: oral bioavailability; T1/2: half-life; Tmax: time to maximal concentration; Vdss, volume of distribution.

A							
Compound	Dose (mg/kg)	N	CLp (ml/min/kg)	Vdss (L/kg)	T1/2 (h)	AUC(0-inf) (hr*ng/mL)	
**14**	3	2	47	0.9	1.3	1,070	
**13**	2	3	1.6	0.3	2.6	21,000	
**7**	3	2	23	1.3	0.8	2,140	
**15**	3	2	156	1.7	0.35	321	

**B**							
Compound	Dose (mg/kg)	N	Cmax (ng/ml)	Tmax (h)	AUC(0-inf) (hrs*ng/mL)	T1/2 (h)	Oral F (%)
**14**	30	3	130	0.5	660	3.8	6
**13**	10	2	17,690	0.75	130,000	4.6	80
**13** (FA)	20	3	2,980	17	260,000	13	80
**7**	20	2	2,500	0.25	7,900	1.6	54
**15**	30	3	18	0.25	30	1.9	1.1

Bold values in this table are referring to the compound identifiers, which are kept in bold throughout the manuscript.

#### 2.3.3 Plasma LPA Profiles in Rat for Compound 13

Male Wistar rats (*n* = 5/group) were dosed orally by gavage with vehicle or 3, 10, and 30 mg/kg of compound **13**, respectively. Plasma samples were collected in triplicates at 0, 1, 3, 6, and 24 h and were added to heparinated tubes and kept at 0°C until further processing. Sampling and immediate processing at 0°C is crucial for the measurements of basal levels, since at higher temperatures, LPA is produced rapidly according to the ATX activity in the sample. One aliquot was used to determine basal levels of LPAs 16:0, 18:0, 18:1, and 20:4 as well as exposure of **13** by LC-MS/MS. A detailed method for LPA analysis is described in the Supplementary Material Section 1.3. The other 2 aliquots were used to determine ATX activity by incubation of the sample at 37°C for 4 h, followed again by analysis of LPA levels in the same way as for the basal LPA levels. LPA profiles were analyzed using MS Excel (Microsoft Corporation).

#### 2.3.4 Oral Treatment with Compound 13

Diets containing compound **13** were manufactured by ssniff Spezialdiäten GmbH (Soest, Germany). Calculations of the daily doses were based on average food intake of rats used in the studies. The regular chow diet for the 60 mg/kg dose was calculated as 8.5 kg and mixed with a solution of **13** in ethanol (4.8%, w/w). The amount of **13** used was 1.0 g per kg of food. After mixing, ethanol was removed in vacuo until dryness. For the preparation of the 20 mg/kg dose, the same procedure was used by applying 0.333 g of **13** per kg of food.

The oral treatment with the ATX-inhibitor **13** (ATX-i; 20 mg/kg; Roche) started 7 days prior to immunization and 3 days prior I/R induction and continued throughout the study. The regular chow diet for the treatment groups was replaced with the same chow diet containing the treatment substance.

#### 2.3.5 Animal Models (EAG, I/R)

For the EAG model, preparation and immunization of the optic nerve antigen (ONA = bovine optic nerve homogenate) was carried out as previously described (Laspas et al., 2011; Joachim et al., 2013). Rats received an intraperitoneal injection with 8 mg/ml ONA. The antigen was mixed with incomplete Freund’s adjuvant (500 µl) plus 3 µg pertussis toxin (both Sigma Aldrich, St. Louis, MO, United States). The animals of the control group were injected with NaCl in Freund’s adjuvant and pertussis toxin. Rats were sacrificed 28 days after immunization.

An established I/R model was used (Schmid et al., 2014; Renner et al., 2017). Therefore, rats were anesthetized with a ketamine/xylazine/vetranquil cocktail (0.65/0.65/0.2 ml). One eye per animal was dilated with 5% tropicamide (Pharma Stulln, Stulln, Germany) and anesthetized topically with conjuncain (Bausch and Lomb, Berlin, Germany). Additionally, a pain medication injection of metamizole (Novalgin; Zentiva, Frankfurt am Main, Germany) was administered subcutaneously. IOP was raised to 140 mmHg for 60 min by elevating a saline reservoir connected to a needle, which was placed into the anterior chamber of the eye. Retinal ischemia was confirmed by observing whitening of the retina via an ophthalmoscope (Mini 300; Heine Optotechnik, Herrsching, Germany). Reperfusion was later reassured by observing the returning blood flow. The contralateral eye was used as a control. Analyses were performed 7 and 14 days after ischemia induction.

#### 2.3.6 IgG Measurement in Aqueous Humor and Serum (EAG)

Aqueous humor and serum were collected at the end of the EAG study. IgG levels were measured in serum and aqueous humor samples from the EAG study (both: 6-7 samples/group) using a sandwich enzyme-linked immunosorbent assay (ELISA) kit (eBioscience, Frankfurt, Germany) according to the manufacturer’s instructions. In brief, samples of aqueous humor from both groups were prediluted 1,000- or 2,000-fold in assay buffer, serum samples were diluted 100,000- or 200,000-fold in assay buffer. 75 µl of assay buffer were inserted into each sample well. 25 µl of the prediluted sample were added to the appropriate wells. All samples were incubated, and the absorbance was read at 405 nm using a microplate reader (AESKU.Reader with Gen5 ELISA Software, AESKU.DIAGNOSTICS, Wendelsheim, Germany) (Reinehr et al., 2018; Tsai et al., 2018).

#### 2.3.7 Intraocular Pressure Measurements (EAG)

The IOP was measured in both eyes of each animal in the EAG study with a rebound tonometer (Tonolab; iCare, Oy, Finland) 1 week before as well as 1, 2, and 3 weeks after immunization (n = 5–6 animals/group). The tonometer was held horizontally in front of the animal’s eye (Pease et al., 2006). 10 measurements per eye were recorded. From these values, the mean value was calculated (Reinehr et al., 2019a).

#### 2.3.8 Electroretinogram Measurements (I/R)

ERG measurements were performed on all eyes 7 and 14 days after I/R induction as previously described (Schmid et al., 2014; Palmhof et al., 2018). Before performing the ERG under dim red light, rats were dark adapted overnight. The readings were done using a full-field flash electroretinograph (HMs ERG system, OcuScience, Henderson, NV, United States). After anesthesia with a ketamine/xylazine cocktail (100/4 mg/kg) eyes were dilated and topically anesthetized. Reference electrodes were placed subcutaneously below the right and left ear and a ground electrode was placed in the base of the tail. Silver thread recording electrodes were placed in the center of the cornea. Scotopic flash ERGs were recorded at 0.1, 0.3, 1, 3, 10, and 25 cd*s/m^2^ for each eye (7 days: 5-6 eyes/group; 14 days: 6-7 eyes/group). Signals obtained from the corneal surface were then amplified, digitized, and averaged using ERG View 4.380R software (OcuScience).

#### 2.3.9 Histology of Retina and Optic Nerve (EAG, I/R)

After explantation, eyes and optic nerves were fixed in 4% paraformaldehyde for 60 min (eyes) or 120 min (optic nerves) and then treated with 30% sucrose overnight. Afterwards, the retinas and optic nerves were embedded in NEG-50 Tissue-Tek medium (Thermo Fisher, Waltham, MA, United States). Cryo-cross sections of the eyes (10 µm) or longitudinal sections of the optic nerves (4 µm) were cut on a microtome (Thermo Fisher), mounted on Histobond Superfrost^+^ slides (Thermo Fisher) and fixed in ice cold acetone for 10 min.

Retinal cross-sections from the I/R studies were stained with hematoxylin and eosin (H&E) to visualize retinal layers and changes in the thickness of the layers using standard protocols (7 days: 5-6 retinas/group; 14 days: 6-7 retinas/group). The thickness of the ganglion cell layer (GCL) was analyzed with the measuring tool of the ZEN2012 image analysis software (Zeiss, Oberkochen, Germany). Therefore, they were measured three times and the value was averaged (Palmhof et al., 2019).

Longitudinal optic nerve sections (EAG: 5-6 nerves/group, I/R 14 days: 6-7 nerves/group) were stained with H&E and luxol fast blue (LFB). Of each staining, three areas per optic nerve section were documented with a microscope equipped with a CCD camera (Axio Imager M1; Zeiss) as previously described (Palmhof et al., 2019). One photo was taken of the proximal part, one of the middle and one of the distal part, right in front of the chiasma.

Cell infiltration in optic nerve sections stained with H&E was evaluated by using a scoring system ranging from 0 to 4 by a masked observer: 0 = no infiltration; 1 = mild cellular infiltration of the optic nerve or optic nerve sheath; 2 = moderate infiltration; 3 = severe infiltration; and 4 = massive infiltration of the optic nerve parenchyma and nodule infiltration (Shindler et al., 2006; Horstmann et al., 2016; Renner et al., 2017). The average score for each optic nerve was used for statistical analysis.

The grade of demyelination was monitored in optic nerve sections stained with LFB. Therefore, staining was scored from 0 to 2 and categorized as follows: 0 = no demyelination; 0.5 = mild demyelination; 1 = moderate demyelination; 1.5 = advanced demyelination; and 2 = severe demyelination up to dissolution of the tissue. The average score for each optic nerve was used for later statistical evaluation (Liu et al., 2014; Horstmann et al., 2016; Wilmes et al., 2018).

#### 2.3.10 Immunohistology of Retina and Optic Nerve (EAG, I/R)

In order to identify certain cell types, specific antibodies were used for immunofluorescence staining in retinas and optic nerves (Casola et al., 2016; Renner et al., 2017; Palmhof et al., 2019). First, the sections were rinsed in phosphate buffered saline solution (PBS). Subsequently, they were blocked and permeabilized with a mixture of serum, 0.1% TritonX-100 and PBS. The primary antibodies (Table 3) were incubated at room temperature overnight followed by incubation with fluorescence labeled secondary antibodies for 1 h (Table 3). In addition, the cell nuclei were stained with DAPI (Serva Electrophoresis, Heidelberg, Germany) for 5 min. Finally, the sections were mounted in Shandon mount media (Thermo Fisher).

**TABLE 3:** Primary and secondary antibodies used for immunohistology and Western Blot analysis of the retina or optic nerve.

Immunohistology antibodies								
Primary antibodies					Secondary antibodies			
Antibody	Company	Catalog number	Tissue	Dilution	Antibody	Catalog number	Company	Dilution
Anti-Brn-3a	Santa Cruz	sc-31984	Retina	1:100	Donkey anti-goat Alexa Fluor 488	705-545-147	Dianova	1:500
Anti-Brn-3a	Santa Cruz	sc-8429	Retina	1:100	Donkey anti-mouse Alexa Fluor 555	ab150106	Abcam	1:500
Anti-cleaved caspase 3	Sigma-Aldrich	C8487	Retina	1:100	Donkey anti-rabbit Alexa 555	A31572	Invitrogen	1:500
Anti-ED1	Millipore	MAB1435	Retina	1:400	Goat anti-mouse Alexa Fluor 488	A21424	Invitrogen	1:500
			Optic nerve	1:200	Goat anti-mouse Alexa Fluor 555	A11029	Invitrogen	1:500
Anti-GFAP	Millipore	AB5541	Retina	1:400	Donkey anti-chicken Cy3	AP194C	Millipore	1:500
			Optic nerve	1:500				
Anti-GLT1	Life Technology	AB1783	Retina	1:500	Donkey anti-guinea pig Alexa Fluor 488	706–545-148	Jackson ImmunoResearch	1:500
Anti-Iba1	Wako	019-19741	Retina	1:400	Donkey anti-rabbit Alexa Fluor 555	A31572	Invitrogen	1:500
			Optic nerve		Goat anti-rabbit Alexa Flour 488	A11008	Invitrogen	1:500
Anti-SMI-32	Covance	SMI-32P	Optic nerve	1:6,000	Goat anti-mouse Alexa Fluor 488	A-11029	Invitrogen	1:400

Western Blot antibodies								
Primary antibodies					Secondary antibodies			
Antibody	Company	Catalog number	Tissue	Dilution	Antibody	Catalog number	Company	Dilution
Anti-β-actin	Sigma-Aldrich	A2228	Retina	1:5,000	Donkey anti-mouse DL800	926-32212	LI-COR	1:20,000
Anti-β-actin	Cell Signaling	4970S	Retina	1:1,000	Donkey anti-rabbit DL800	SA5-10044	Thermo Fisher	1:20,000
Anti-β-III-tubulin	R&D Systems	MAB1195	Retina	1:15,000	Donkey anti-mouse Alexa Fluor 680	A10038	Invitrogen	1:5,000
Anti-GFAP	Millipore	AB5541	Retina	1:3,000	Donkey anti-chicken IR680RD	925-68075	LI-COR	1:20,000
Anti-Iba1	SySy	234,003	Retina	1:1,000	Donkey anti-rabbit Alexa Fluor 680	A10043	Invitrogen	1:5,000

Six retinal or optic nerve sections were used for each staining. In the EAG study, 5-6 retinas/group as well as 5-6 nerves/group were included in the evaluation. Regarding the I/R studies, in the I/R 7 days study 5-6 retinas/group were included, while the I/R 14 days study consisted of 6-7 retinas/group and 5-6 nerves/group. All sections were examined with a fluorescence microscope (Axio Imager M1). Photographs were taken in two central and two peripheral areas of the retina. In the optic nerve, three photographs were captured (proximal, middle, central). In general, the same camera setting, including exposure time, was applied on all images obtained for one staining protocol. Then, these photographs were masked and cut with a predefined window (Corel PaintShop Pro, Ottawa, ONT, Canada). For each retinal or optic nerve field, the number of Brn-3a^+^, cleaved caspase 3^+^, Iba1^+^, and ED1^+^ labeled cell bodies were counted using ImageJ Software (NIH; Bethesda, MD, United States). In regard to ED1^+^ cells, only the one colocalized with Iba1^+^ ones were included in the analysis.

Measurement and analysis of GFAP labeled areas in the retina was performed using ImageJ software with established protocols (Palmhof et al., 2019). Briefly, images were transformed into grayscale (32-bit). To minimize interferences with background labeling, a rolling ball radius of 50 pixels for EAG and I/R was subtracted. Then, for each picture a suitable lower threshold was determined. The ideal threshold can be obtained when the greyscale picture corresponds with the original one. After all lower thresholds from each picture were obtained, the mean value was calculated, and this number was then used for the final analysis. The percentage of the labeled area was then measured between these defined thresholds (EAG: lower threshold: 8.87; upper threshold: 85.00; I/R: lower threshold: 9.82; upper threshold: 165.00).

The optic nerve neurofilament (SMI-32) evaluation was based on a scoring system: 0 = intact structure; 1 = shortened axons, neurofilament swellings, building of retraction bulb; and 2 = loss of structural integrity, fissures, many retraction bulbs (Schlamp et al., 2006; Wang et al., 2011; Reinehr et al., 2018).

The GFAP signal in the optic nerve was also evaluated based on a scoring system: 0 = parallel arranged GFAP structure, less ramifications; 1 = strong and mostly organized gliosis, beginning of destruction; and 2 = loss of structure, strong, and disorganized ramification.

#### 2.3.11 Western Blot Analysis of Retinal Tissue (I/R)

In order to quantify the protein level in I/R retina samples at 7 and 14 days, Western Blot analysis were performed (Casola et al., 2016). First, retinas (7 days: 5 retinas/group; 14 days: 5-6 retinas/group) were lysed in RIPA buffer (Cell Signaling Technology, Frankfurt, Germany) plus protease inhibitor (Sigma-Aldrich). Then, the protein concentration for each sample was determined using a BCA-kit. 10 µg protein was pipetted into each SDS-gel lane (NuPAGE, Invitrogen, Carlsbad, CA, United States) for electrophoresis (50 min, 200 V). Afterwards, the proteins were transferred onto a nitrocellulose membrane and blotted for 60 min at 200 V (Wet Blotter; X Cell sure Lock, Invitrogen). The same primary antibodies as used for immunohistology (Table 3) were applied to detect the protein expressions of ganglion cell and glia markers. Fluorescence labeled secondary antibodies were applied and detected using an Odyssey Infrared Imaging System (LI-COR Biosciences, Lincoln, NE, United States). Band intensities were compared between groups.

#### 2.3.12 Statistical Analysis (EAG, I/R)

Data are presented as mean ± SD, unless noted otherwise. Regarding the *in vivo* data, groups were compared by ANOVA, followed by Tukey post-hoc test (Statistica; V 13; Dell, Tulsa, OK, United States). *p*-values below 0.05 were considered to be statistically significant. ONA/I/R vs. control and ONA + ATX-i/I/R + ATX-i vs. control: *p < 0.05, **p < 0.01, ***p < 0.001. ONA + ATX-i/I/R + ATX-i vs ONA/I/R: ^#^ *p* < 0.05, ^##^ *p* < 0.01, ^###^ *p* < 0.001.

## 3 Results

### 3.1 Design, Synthesis, and Characterization of ATX-Inhibitors

#### 3.1.1 Synthetic Approach

A representative synthesis of an ATX-inhibitor with a new central core is shown in Figure 1B for compound **7**. Similar approaches were used for the other compounds (Supplementary Material Section 1.1, S1.1–S1.13 for procedures and analytical data). Synthesis of compound **7** started from enantiomerically pure di-carboxylic acid ester **20** (Rodríguez Sarmiento et al., 2003). Diester **20** was reduced to di-methanol **21** with LiBH_4_ in THF, followed by mesylation in the presence of methanesulfonyl chloride and NEt_3_ to afford **22** in high yield over two steps. Treatment of **22** with phenylmethanamine and potassium carbonate in CH_3_CN at 95°C for 24 h gave the BOC-protected bicyclic intermediate **23**. De-benzylation of **23** was achieved by catalytic hydrogenation with palladium on charcoal in MeOH at RT to afford the desired bicyclic building block **24**. This material was coupled with commercially available *1H*-benzotriazole-5-carboxylic acid (**25**) in the presence of HATU and NMM in DMF at RT in high yield (83%) to provide **26** which was subsequently de-protected with HCl in ^i^PrOH (∼5 M) at RT, affording HCl salt **27**. Then, [3,5-dichlorophenyl]methanol was treated with CDI in CH_3_CN at RT followed by addition of **27** and Net_3._ This mixture was heated to reflux for 16 h to provide the desired compound **7** in 68% yield and an excellent purity of 99% after purification.

#### 3.1.2 Modification of the Core and the ZBG

Our initial goal was the replacement of the piperidine core of **1** with different bicyclic and spirocyclic cores, resulting in removal of the basic nitrogen and the potentially labile β-aminocarbonyl structure. Initially, both the ZBG and the lipophilic pocket substituent were kept constant to allow direct comparison of the new inhibitors with **1**. Besides activity, lipophilicity (LogD), solubility and *in vitro* metabolic stability against human, rat, and mouse microsomes (Table 1: column CL h/m/r), an additional key parameter that was investigated was the formation of reactive intermediates after metabolic activation followed by trapping and adduct formation with glutathione (Table 1: column GSH adducts). It was found that for example introduction of a trans octahydro-pyrrolo-pyridine core was possible without loss of activity (compound **2**), however, lipophilicity increased significantly, solubility remained low and formation of GSH adducts was still observed. Similar observations were made when spirocyclic building blocks were introduced as central cores. Compound **4** retained good activity, while the different orientation of the core in **3** led to significant loss of activity. In both cases, LogD increased, and solubility remained low without a significant gain in metabolic stability *in vitro* against microsomes. Introduction of a *cis*-octahydropyrrolopyrrole core (compound **5**) led to loss of activity, while a slight increase in solubility was observable. With all new derivatives **3**, **4**, and **5** GSH adducts were still observed. It became clear from these early data that additional changes to both the ZBG and the lipophilic pocket binder (represented by the dichlorobenzyl moiety in **1**–**5)** were required.

The next step was modification of the ZBG, which was done in a similar way as described by others (Kuttruff et al., 2017). For this change, the benzotriazole ZBG was found to be particularly interesting. Hence, this ZBG was combined with several of the most promising cores and results are shown in Table 1 with compounds **6**–**10**. Introduction of this ZBG resulted in a minimally reduced or equal lipophilicity, and a minor trend towards better solubility. The best potency was achieved with the *cis*-decahydropyrroloazepine core (compound **10**), however, this core resulted in a markedly reduced metabolic stability. In contrast, the most balanced potency and property profile was observed with the *trans*-octahydropyrrolopyrrole, which also showed the most promising metabolic stability. However, since all compounds **6**–**9** retained the GSH flag, it became clear that additional changes to the dichlorobenzyl portion of the molecule were necessary.

#### 3.1.3 Modification of the Lipophilic Pocket Residue

Several lipophilic pocket residues were investigated, and a set of representative compounds is shown in Table 4. Cinnamic acid derivatives such as **11** showed a high potency, a reduced lipophilicity and improved metabolic stability, but solubility turned out to be very low. Interestingly, a shift of the oxygen such as in compound **12** resulted in a significant loss of activity. Attempts to introduce some more polar functional groups were partially successful: Methylsulfone **16** retained a decent potency with the expected reduction of LogD, markedly improved solubility and better metabolic stability, but these improvements were accompanied by a significant loss in permeability. Compounds **13** and **14** showed the most balanced overall profile with decent potency and acceptable LogD, solubility, permeability and *in vitro* metabolic stability against microsomes. For compound **15**, most parameters were also promising, but the permeability was considerably lower. Notably, all examples in Table 4 did not show a potential for reactive metabolites and no GSH adducts could be observed any more. This was in line with the understanding that in **1** both the ZBG and the dichlorobenzyl moiety were prone to metabolic activation, which is supported by the GSH data given in Table 1.

**TABLE 4:** Data of ATX-inhibitors with benzotriazole ZBG and *trans*-octahydropyrrolopyrrole core. A clearance value of 10 μL/m/mg prot indicates a low microsomal clearance, which is essentially below the detection limit of the assay.

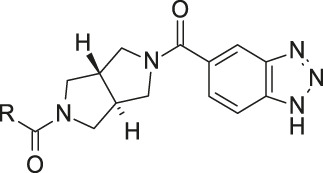	
Cmpd	R	hATX IC_50_ [nM]	LogD (pH 7.4)	Solubility [µg/mL], pH 6.5	Permeability Pe [10^–6^ * cm/s]	Mic CL h/m/r [µL/m/mg prot]	GSH adducts
**11**	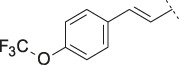	1	2.3	<1	*Na*	10/10/*na*	Neg
**12**	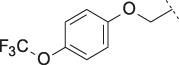	137	3.2	8	1.8	10/*na*/10	Neg
**13**	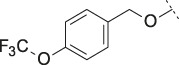	6	3.2	28	4.5	10/10/10	Neg
**14**	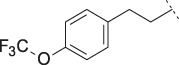	6	2.5	46	2.7	10/27/40	Neg
**15**	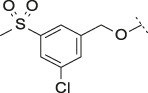	8	2.0	190	0.4	10/10/*na*	Neg

Na = not available.

#### 3.1.4 X-Ray Co-crystal Structures of Compounds 1, 10, and 13

In order to confirm the binding mode of the novel ATX-inhibitors with different cores and modified ZBGs, selected compounds were soaked into apo-crystals of rat ATX and their structures were determined by X-ray crystallography. Procedures and data for all X-ray structures are provided in the Supplementary Material Section 1.2 and Supplementary Table S1. The structures for **10** (Figure 1C) and **13** (Figure 1D) showed an identical binding mode as the one determined for the seed compound **1**, with an interaction of the ZBG with the active site zinc ion and a good fit to the lipophilic pocket. In all cases, the oxygen of the carbonyl group of the carbamate forms a hydrogen bond to the backbone NH of Trp 275. These structural data also indicated that introduction of several bicyclic cores was possible as long as the exit vectors would still allow the ZBG to interact with the zinc ion and ensure proper fit to the lipophilic pocket in a low energy conformation. In case of **10**, a cis fusion between the 5-membered and the 7-membered rings of the core is tolerated, whereas in the case of **13** a trans fusion of the two 5-membered pyrrolidine rings was preferred over a cis fusion as suggested by compound **8**, which was considerably less potent (Table 1).

### 3.2 Evaluation of *in vivo* PK and LPA Modulating Properties

A set of representative compounds with the most promising *in vitro* data was investigated with regard to pharmacokinetic properties *in vivo* in rat at a single dose (compounds **7**, **13**, **14**, and **15**; Tables 2A,B). The main goal of the PK analysis was to investigate, if *in vitro* microsomal stability would translate into sufficient exposure *in vivo*. As the number of animals for the individual PK experiments was kept low, a statistical comparison between the individual compounds was not attempted. While for examples **7**, **14**, and **15** insufficient *in vivo* exposures were reached at the indicated doses, compound **13** showed sufficient oral bioavailability and low plasma clearance *in vivo* as well as a suitable half-life to allow for bid or food admix dosing. Hence, these data indicated that **13** was a suitable candidate for further PK/PD assessment *in vivo*.

**FIGURE 2:**
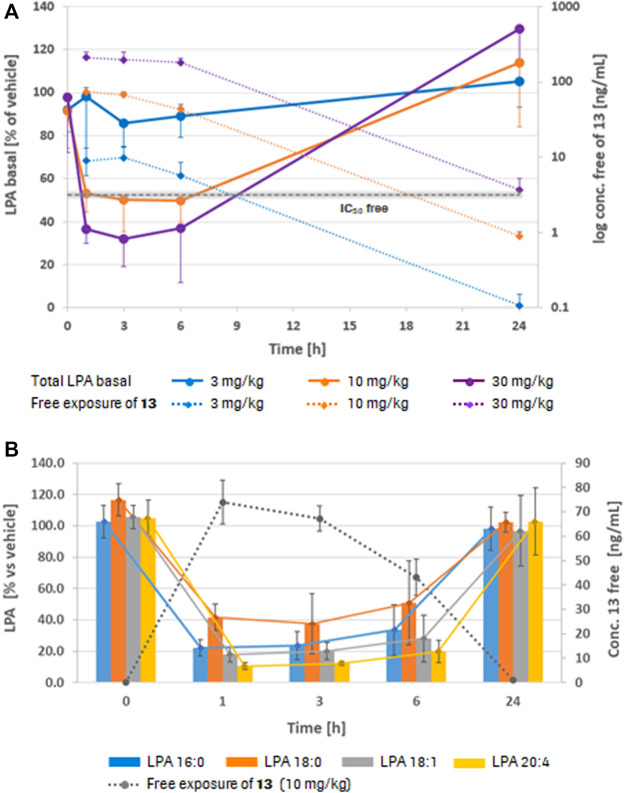
Plasma LPA profiles in rat (PK/PD). **(A)** Dose and free exposure dependent lowering of total basal plasma LPA *in vivo* in rat at 1, 3, 6, and 24 h after dosing with **13**, normalized to the vehicle control. For all doses (3, 10, and 30 mg/kg po), 5 animals/group were used. **(B)** Inhibition of the formation of 4 different LPA species in rat plasma after a single oral dose of **13** (10 mg/kg, n = 5) at the same time points, normalized to the vehicle control (LPA formation was measured after incubation of the plasma samples at 37°C for 2 h). Values are mean ± SD.

Compound **13** was then used in an acute PK/PD experiment in rats at 3 doses (3, 10, and 30 mg/kg po, qd; n = 5/group) and plasma levels of 4 different LPA species (LPA 16:0, 18:0, 18:1, and 20:4) were determined by LC/MS. For **13**, a dose dependent plasma exposure resulted in a dose dependent lowering of total plasma LPA (as the sum of the 4 LPA species). Basal levels of LPA 16:0, 18:0, and 18:1 were quite low (approx. 10, 4, and 8 nM, respectively) and relatively close to the detection limit of the LC/MS method (approx. 2 nM), whereas LPA 20:4 had a higher basal concentration of about 84 nM. Therefore, modulation of total basal LPA was largely driven by modulation of LPA 20:4 (Figure 2A). An IC_50_ of approx. 3.2 ng/ml (free plasma concentration) was determined for the lowering of total basal LPA.

To better observe the modulation of the individual LPA species, a second set of plasma samples was incubated at 37°C for 2 h prior to LC/MS analysis. Due to continued ATX activity in the incubated plasma, LPA levels (LPA 16:0: 1,600 nM; 18:0: 800 nM; 18:1: 900 nM; and 20:4: 6,500 nM, respectively) turned out substantially higher through conversion of plasma LPC to LPA. Any differences in LPA levels observed after treatment with **13** can therefore be considered as a result of inhibition of plasma ATX. Figure 2B shows modulation of the four different LPA species after plasma incubation, indicating that after a single dose of **13** of 10 mg/kg, formation of all four LPA species were significantly inhibited for 6 h and more. However, no inhibition of LPA formation was visible 24 h post-dose. This is in line with the plasma exposures of **13** at the different time points (dotted line). Data for all LPA species resulting from plasma incubations indicated a lower IC_50_ of about 0.7 ng/ml (free concentration) for **13** in blood. Compound **13** was then further investigated in a food admix PK at a dose of ∼20 mg/kg per day, based on average body weight and normal food consumption which suggested that this dose should result in efficient LPA modulation *in vivo* based on the AUC. Results of this study are summarized in Table 2B.

### 3.3 *In vivo* Studies

#### 3.3.1 Effects of ATX-Inhibitor Treatment in the EAG Model

##### No Alterations in Intraocular Pressure

The IOP was measured 1 week before as well as 1, 2, and 3 weeks after the immunization in all groups. One week before immunization, the IOP was unaltered in ONA animals (10.34 ± 0.67 mmHg) compared to controls (10.36 ± 1.03 mmHg; *p* = 0.999). Also, no alterations were observed in ONA + ATX-i treated animals (10.91 ± 0.50 mmHg; *p* = 0.521). At 1 week, IOP remained unchanged in the ONA group (10.44 ± 0.62 mmHg) in comparison to controls (11.26 ± 0.54 mmHg; *p* = 0.058). No changes were detected in the ONA + ATX-i group compared to controls (10.54 ± 0.40 mmHg; *p* = 0.142). At 2 weeks, the IOP remained unaltered in ONA (10.74 ± 0.55 mmHg; *p* = 0.119) as well as in ONA + ATX-i groups (10.85 ± 0.42 mmHg; *p* = 0.276) compared to controls (11.36 ± 0.52 mmHg). No changes in the IOP were observed in ONA (10.77 ± 0.77 mmHg; *p* = 0.205) and ONA-ATX-i animals (10.69 ± 0.60 mmHg; *p* = 0.197) in comparison to controls (11.47 ± 0.62 mmHg) 3 weeks after immunization (Supplementary Figure S1).

##### Altered IgG Levels After ATX-i Treatment

**FIGURE 3:**
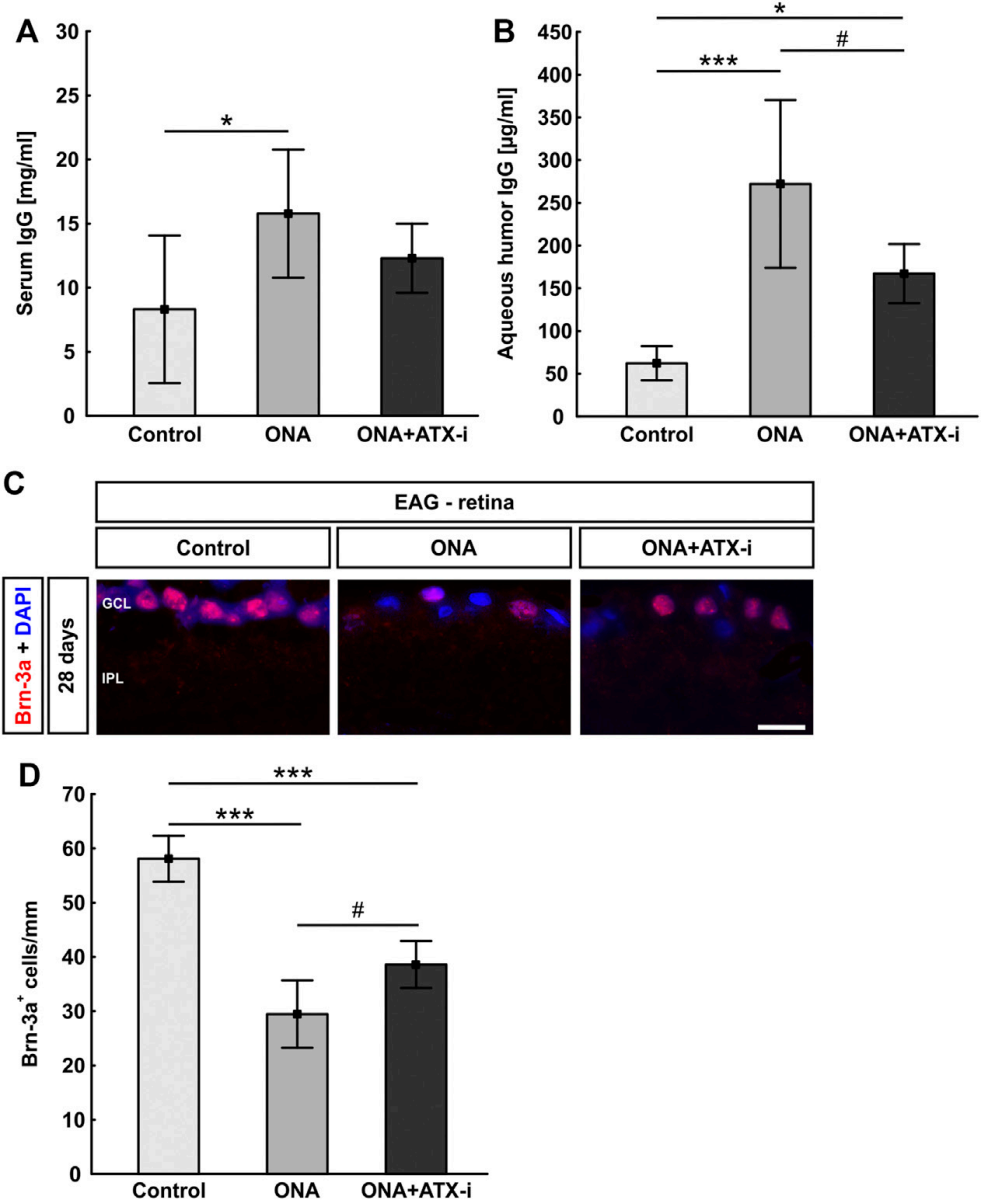
Inhibited immune response and RGC loss in EAG. **(A)** Serum IgG levels were only increased in the ONA group, but not in ONA + ATX-i animals compared to controls. **(B)** IgG levels in the aqueous humor were significantly enhanced in ONA and ONA + ATX-i animals. However, in the ONA + ATX-i group, a lower IgG concentration was observable when compared to ONA samples. **(C)** RGCs were labelled with an antibody against Brn-3a (red), while cell nuclei were counterstained with DAPI (blue). **(D)** Significantly fewer RGCs were noted in ONA and ONA + ATX-i retinas compared to controls. Nevertheless, significantly more Brn-3a^+^ RGCs were detected in ONA + ATX-i animals compared to ONA ones. Values are mean ± SD. 6–7 samples/group. Abbreviations: GCL = ganglion cell layer; IPL = inner plexiform layer. Scale bar: 20 μm *p < 0.05; ***p < 0.001; ^#^ *p* < 0.05.

After ONA immunization, serum IgG levels were significantly increased compared to controls (ONA: 15.78 ± 5.00 mg/ml; control: 8.31 ± 5.76 mg/ml; *p* = 0.024). After ATX-i treatment, no significant differences were observed in ONA + ATX-i animals (12.30 ± 2.70 mg/ml) in comparison to the control group (*p* = 0.339; Figure 3A). In the aqueous humor, IgG levels were also significantly enhanced in the ONA (272.24 ± 98.17 μg/ml; *p* < 0.001) as well as in the ONA + ATX-i group (167.26 ± 34.50 μg/ml; *p* = 0.025) compared to controls (62.31 ± 19.91 μg/ml). However, ONA + ATX-i animals displayed a significantly lower IgG concentration in the aqueous humor compared to the ONA group (*p* = 0.025; Figure 3B).

##### Mild Protection of Retinal Ganglion Cells and Less Glial Cell Response

Brn-3a^+^ RGC numbers were significantly decreased in ONA animals (29.47 ± 6.20 cells/mm) compared to controls (58.11 ± 4.22 cells/mm; *p* < 0.001). Furthermore, significantly fewer Brn-3a^+^ cells were detected in ONA + ATX-i retinas in comparison to the control group (38.59 ± 4.33 cells/mm; *p* < 0.001). However, a higher number of Brn-3a^+^ RGCs was noted in ONA + ATX-i animals when compared to the ONA group (*p* = 0.032; Figures 3C,D), suggesting a mild protective effect on RGCs through ATX-i treatment.

**FIGURE 4:**
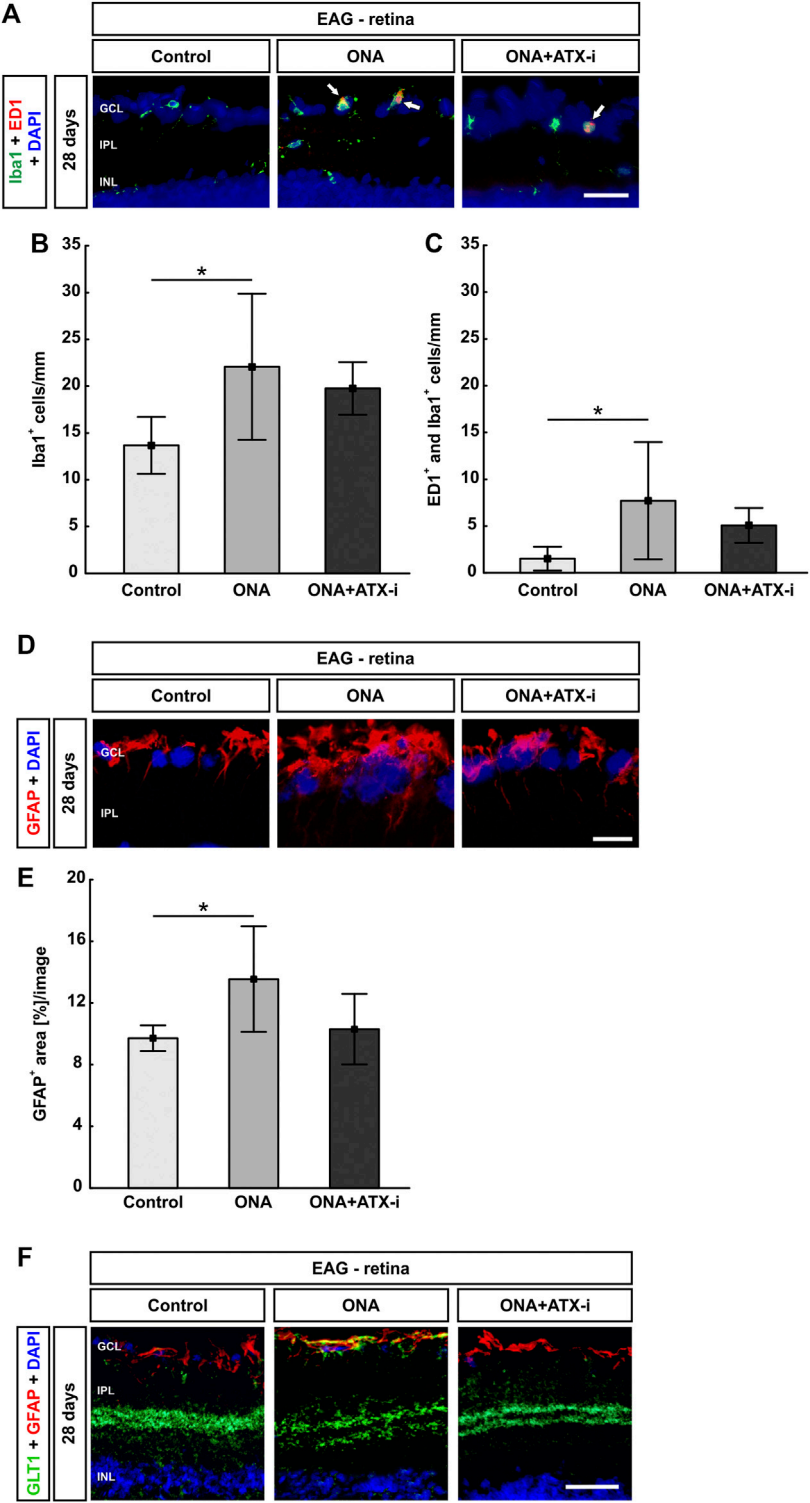
Less glial response in EAG retina. **(A)** The total number of microglia was stained with an antibody against Iba1 (green) and activated microglia were additionally marked with ED1 (red, arrows). DAPI counterstained cell nuclei (blue). **(B)** Iba1^+^ cell numbers were significantly increased in ONA retinas, but not in ONA + ATX-i animals, compared to controls. **(C)** Furthermore, significantly more activated microglia were only detected in the ONA group. No alterations could be observed in ONA + ATX-i retinas compared to controls. **(D)** Retinal cross-sections were stained with an antibody against GFAP (red; astrocytes). DAPI (blue) labelled cell nuclei. **(E)** The GFAP^+^ area was significantly increased in ONA retinas, but not in ONA + ATX-i animals compared to controls. **(F)** Retinal cross-sections were co-labelled with antibodies against GFAP (red) and GLT1 (green), while DAPI counterstained cell nuclei (blue). In control as well as in ONA + ATX-i retinas, GLT1 was predominantly observed in the inner plexiform layer. In contrast, enhanced staining patterns in the nerve fiber and GCL, in co-localization with GFAP, could be noted in the ONA group. Values are mean ± SD. 5-6 retinas/group. Abbreviations: GCL = ganglion cell layer; IPL = inner plexiform layer; INL = inner nuclear layer. Scale bars: 20 μm *p < 0.05.

Iba1^+^ microglia cell counts were significantly increased in ONA animals (22.06 ± 7.81 cells/mm) compared to controls (13.67 ± 3.04 cells/mm; *p* = 0.037). No changes were noted in the ONA + ATX-i retinas when compared to controls (19.76 ± 2.82 cells/mm; *p* = 0.192; Figures 4A,B). Significantly more ED1^+^ and Iba1^+^ microglia were observed in the ONA group (7.70 ± 6.27 cells/mm; *p* = 0.042), but not in the ONA + ATX-i group (5.08 ± 1.86 cells/mm), compared to controls (1.52 ± 1.28 cells/mm; *p* = 0.354; Figures 4A,C).

The area analysis of GFAP revealed a larger area in ONA retinas (13.55 ± 3.42 area [%]/section) in comparison to controls (9.72 ± 0.84 area [%]/section; *p* = 0.041). No changes could be noted in the ONA + ATX-i group (10.30 ± 2.29 area [%]/section; *p* = 0.924; Figures 4D,E).

Additionally, GFAP was co-labeled with an antibody against GLT1, a marker for protoplasmic astrocytes (Velmeshev et al., 2019). In control as well as in ONA + ATX-i retinas, GLT1 was predominantly observed in the inner plexiform layer. In contrast, enhanced staining patterns in the nerve fiber and GCL in co-localization with GFAP could be noted in the ONA group (Figure 4F).

##### Only Slight Optic Nerve Neurofilament Protection

**FIGURE 5:**
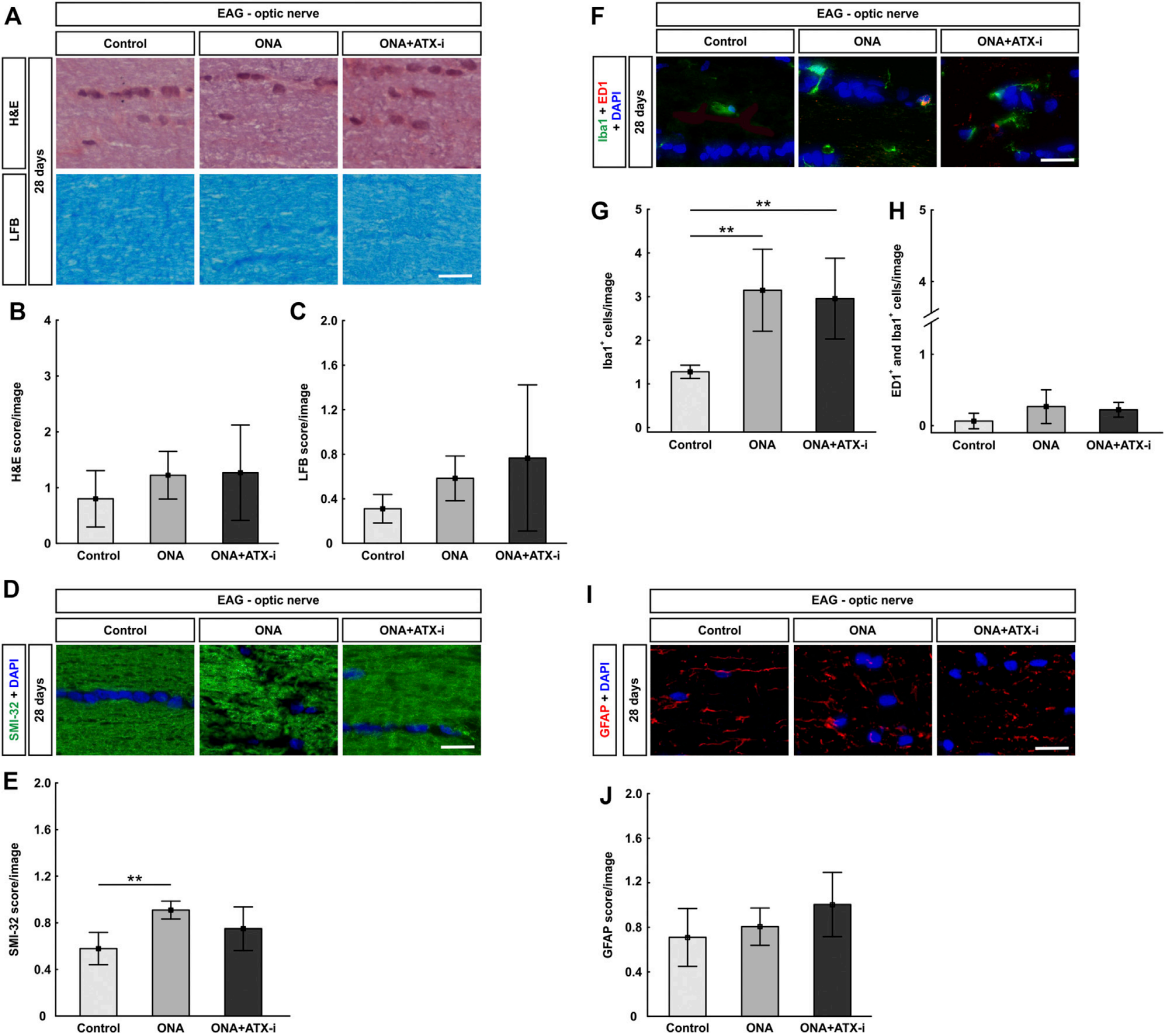
Mild protection of EAG optic nerves. **(A)** Optic nerves were stained with H&E and LFB. **(B)** The H&E score revealed no signs of inflammation within all groups. **(C)** Also, no disruption of myelin could be observed via the LFB score in all groups. **(D)** The neurofilament was labelled with an antibody against SMI-32 (green) and DAPI stained cell nuclei (blue). **(E)** A disruption of neurofilaments could be detected in ONA optic nerves via SMI-32 score. No changed were noted in ONA + ATX-i animals compared to controls. **(F)** The total number of microglia was labelled with an antibody against Iba1 (green) and activated microglia were additionally stained with ED1 (red). DAPI counterstained cell nuclei (blue). **(G)** Significantly more Iba1^+^ microglia were noted in ONA and in ONA + ATX-i optic nerves. **(H)** However, no alterations in regard to active microglia were noted within all groups. **(I)** Astrocytes were stained against anti-GFAP (red), while DAPI labelled cell nuclei (blue). **(J)** The GFAP score in ONA as well as ONA + ATX-i optic nerves was not altered in comparison to control ones. Values are mean ± SD. 5-6 nerves/group. Scale bars: 20 μm **p < 0.01.

The optic nerve H&E score showed no signs of inflammation in the ONA (1.22 ± 0.43; *p* = 0.536) as well as in ONA + ATX-i group (1.27 ± 0.86; *p* = 0.470) compared to controls (0.80 ± 0.51; Figures 4A,B). LFB scores, representing myelination, revealed no alterations between ONA animals (0.58 ± 0.20; *p* = 0.531) and control ones (0.31 ± 0.13). Furthermore, no differences were noted in ONA + ATX-i optic nerves (0.77 ± 0.66; *p* = 0.196; Figures 5A,C).

SMI-32 labeling showed a significant higher score in the ONA group (0.91 ± 0.08) compared to controls (0.58 ± 0.14; *p* = 0.003). This indicates a disruption of neurofilaments. However, no alterations were noted in ONA + ATX-i optic nerves (0.75 ± 0.19; *p* = 0.158; Figures 5D,E), suggesting a protective effect by ATX-i.

A significant increase of Iba1^+^ microglia was noted in the ONA (3.14 ± 0.94 cells/image; *p* = 0.004) as well as in the ONA + ATX-i group (2.96 ± 0.92 cells/image; *p* = 0.009) compared to controls (1.28 ± 0.15 cells/image; Figures 4F,G). However, alterations in ED1^+^ and Iba1^+^ active microglia numbers were detected neither in the ONA (0.27 ± 0.24 cells/image; *p* = 0.148) nor in the ONA + ATX-i optic nerves (0.22 ± 0.10 cells/image; *p* = 0.292) compared to controls (0.06 ± 0.11 cells/image; Figures 5F,H).

The GFAP, labelling astrocytes, score in the optic nerves revealed no differences in ONA animals (0.81 ± 0.17; *p* = 0.802) compared to controls (0.71 ± 0.26). Also, no changes were detectable in the ONA + ATX-i group (1.00 ± 0.29; *p* = 0.163; Figures 5I,J).

#### 3.3.2 Effects of Compound 13 (ATX-I) in the I/R Model

##### Mild Protection of Retinal Function and Thickness After ATX-I Treatment

**FIGURE 6:**
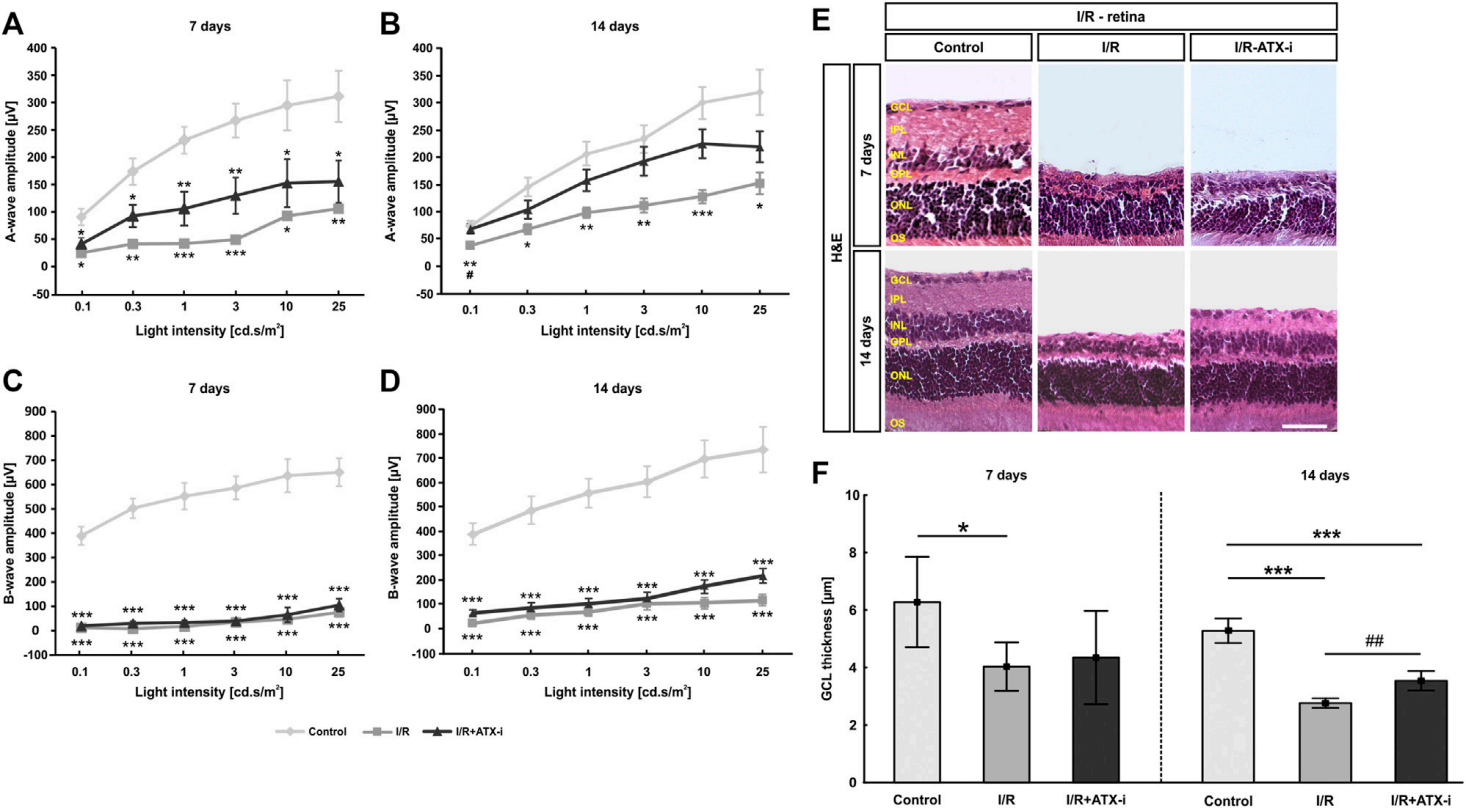
Moderate effect on I/R retinas. **(A)** After 7 days, recordings of a-wave amplitudes exhibited significant differences between the control and both ischemic groups. **(B)** 14 days after ischemia induction, a-wave amplitudes of the ischemic group were significant lower when compared to control eyes. Nevertheless, the amplitude reduction in the ATX-I group was less prominent. **(C)** The I/R and the I/R + ATX-i group revealed significantly lower b-wave amplitudes than the control group 7 days after I/R. **(D)** 14 days after ischemia, a strong decline of the b-wave amplitudes was detected in both ischemic groups. **(E)** Staining with H&E was performed 7 as well as 14 days after ischemia induction to visualize the morphology of the retinal layers. **(F)** At 7 days, the GCL thickness was significantly thinner in I/R retinas compared to control ones. The thickness of the GCL was significantly reduced in the I/R and the I/R + ATX-i group compared to controls at 14 days. However, a broader GCL thickness was revealed in I/R + ATX-i retinas when compared to the I/R group. Values are mean ± SD. **(A,B)**: 5–6 eyes/group; **(C,D)**: 6–7 eyes/group; **(E)**: 5–6 retinas/group; **(F)**: 6–7 retinas/group. Abbreviations: GCL = ganglion cell layer; IPL = inner plexiform layer; INL = inner nuclear layer; OPL = outer plexiform layer; ONL = outer nuclear layer; OS = outer segment. Scale bar: 20 μm *p < 0.05; **p < 0.01; ***p < 0.001; ^#^ *p* < 0.05; ^##^ *p* < 0.01.

7 days after ischemia induction, a significant decrease in the a-wave amplitude was noted in the I/R and the I/R + ATX-i group in comparison to the control group at all measured light intensities (*p* < 0.050; Figure 6A). After 14 days, a significant reduction of the a-wave amplitude was detected in ischemic eyes in comparison to control ones at all light intensities (*p* < 0.050). The a-wave amplitude of the I/R + ATX-i group was comparable to the control ones at all light intensities (*p* > 0.050). When compared to the I/R group, a significant increase in the a-wave amplitude was measurable in the I/R + ATX-i group at 0.1 cd s/m^2^ (*p* = 0.027; Figure 6B).

A significantly lower b-wave amplitude was revealed in all I/R and I/R + ATX-i eyes at all investigated light intensities 7 days after ischemia (*p* < 0.001; Figure 6C). At 14 days, the b-wave amplitude was significantly declined in both I/R and I/R + ATX-i eyes compared to control ones at all measured light intensities (*p* < 0.001; Figure 6D).

In relation to the control retinas (6.28 ± 1.57 µm), a significant reduction of the thickness of the GCL was measured after 7 days in the I/R (4.03 ± 0.84 µm; *p* = 0.034), but not in the I/R + ATX-i group (4.34 ± 1.63 µm; *p* = 0.100; Figures 6E,F). Regarding the 14 days point in time, the GCL was significantly thinner in the I/R (2.76 ± 0.16 µm; *p* < 0.001) and in I/R + ATX-i retinas (3.54 ± 0.34 µm; *p* < 0.001) compared to control ones (5.28 ± 0.43 µm). However, a significant broader thickness of the GCL was evaluated in the I/R + ATX-i group against the I/R group (*p* = 0.003; Figures 6E,F).

##### Mild Protection of Retinal Ganglion Cells

**FIGURE 7:**
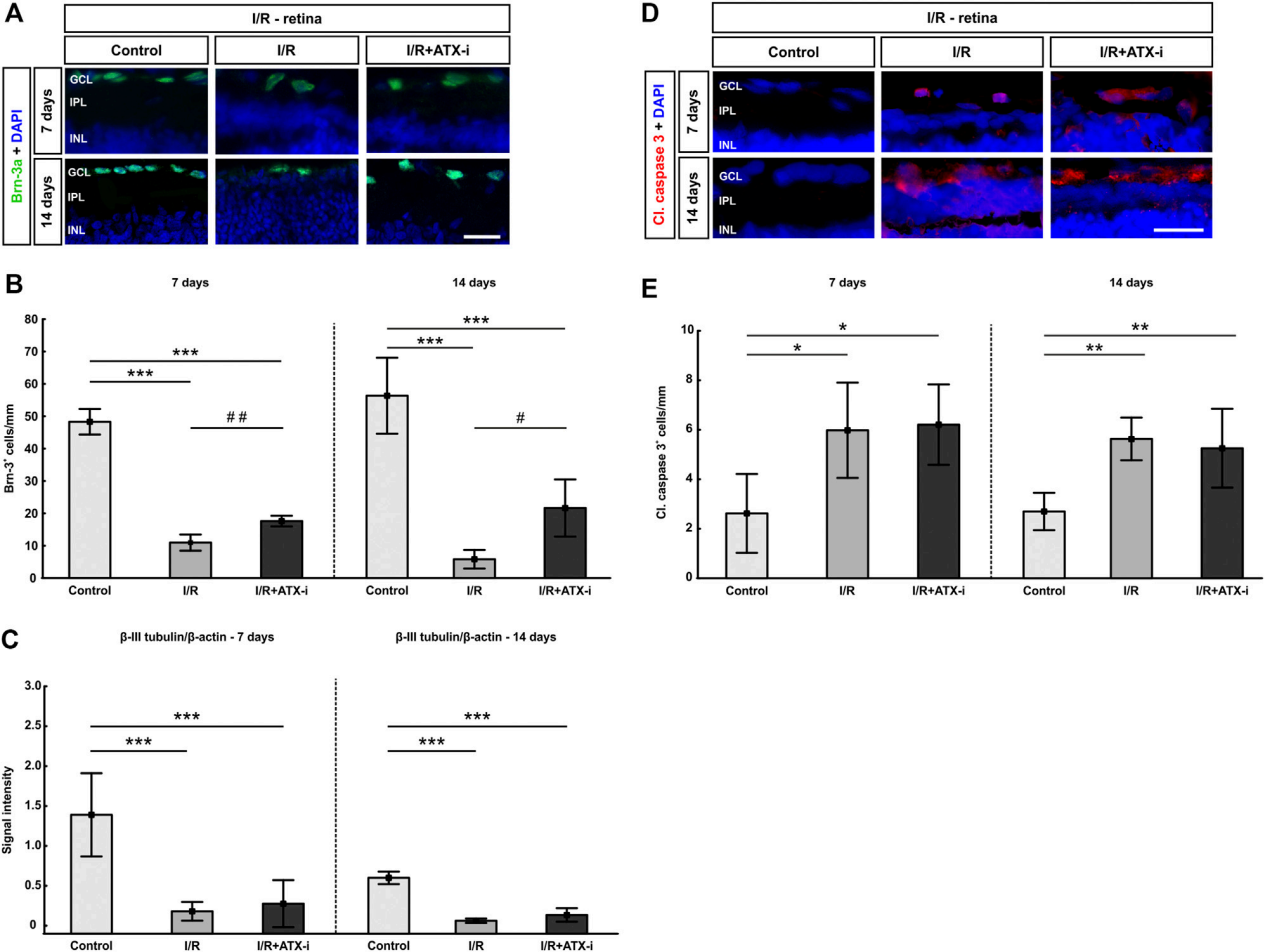
Protection of retinal ganglion cells. **(A)** Retinal cross-sections stained with anti-Brn-3a (green) to label retinal ganglion cells at 7 and 14 days post I/R. Cell nuclei were visualized with DAPI (blue). **(B)** At both points in time, a significant lower number of RGCs was detectable in the I/R group and the IR + ATX-i group. However, significantly more RGCs were counted in the I/R + ATX-i group in comparison to the I/R group. **(C)** Western Blot analysis of retinal samples revealed a significantly less β-III-tubulin in both ischemic groups, the I/R and IR/+ATX-i group, at both points in time when compared to the control group. **(D)** Immunostaining of apoptotic cells with cleaved caspase 3 (red) after 7 and 14 days. Cell nuclei were labelled with DAPI (blue). **(E)** The number of cleaved caspase 3^+^ cells was significantly higher in I/R and I/R + ATX-i retinas compared to controls at 7 and 14 days. Values are mean ± SD. 7 days: 5–6 retinas/group; 14 days: 6–7 retinas/group. Abbreviations: GCL = ganglion cell layer; IPL = inner plexiform layer; INL = inner nuclear layer. Scale bars = 20 μm *p < 0.05; **p < 0.01; ***p < 0.001; ^#^ *p* < 0.05; ^##^ *p* < 0.01.

In comparison to the control group (48.29 ± 3.94 cells/mm), a significant RGC loss was observed 7 days post ischemia in the I/R group (10.95 ± 2.48 cells/mm; *p* < 0.001) and in the I/R + ATX-i group (17.61 ± 1.63 cells/mm; *p* < 0.001). However, significantly more Brn-3a^+^ cells were noted in I/R + ATX-i retinas compared to I/R ones (*p* = 0.008), pointing towards a protective effect of ATX-i (Figures 7A,B). The protective effect on RGCs was retained 14 days after ischemia. Here a significant RGC loss was detected in the I/R group (5.81 ± 2.85 cells/mm; control: 56.32 ± 11.74 cells/mm; *p* < 0.001) and in the I/R + ATX-i group (21.62 ± 8.84 cells/mm; *p* < 0.001). However, a significant difference was noted between the I/R and the I/R + ATX-i group (*p* = 0.017), with a higher number of Brn-3a^+^ RGCs in ATX-i treated eyes (Figures 7A,B).

Western Blot analysis revealed a significant decrease in β-III tubulin protein levels in the I/R (0.18 ± 0.17) and the I/R + ATX-i (0.28 ± 0.30) group compared to control retinas (1.39 ± 0.52; both: *p* < 0.001; Figure 7C) at 7 days. 14 days after I/R, the β-III-tubulin levels of both I/R groups (I/R: 0.06 ± 0.03 and I/R + ATX-i: 0.13 ± 0.08) were also significantly decreased compared to control ones (0.60 ± 0.08; both: *p* < 0.001; Figure 7C).

The number of cleaved caspase 3^+^ apoptotic RGCs was significantly increased in I/R (5.97 ± 1.93 cells/mm; *p* = 0.012) and I/R + ATX-i retinas (6.21 ± 1.62 cells/mm; *p* = 0.014) compared to controls (2.62 ± 1.59 cells/mm) at 7 days (Figures 7D,E). Also 14 days after I/R, significantly more cleaved caspase 3^+^ cells were observed in I/R (5.38 ± 0.83 cells/mm; *p* = 0.001) and I/R + ATX-i animals (5.02 ± 1.52 cells/mm; *p* = 0.002) when compared to control ones (2.58 ± 0.72 cells/mm; Figures 7D,E).

##### Fewer Microglia Cells and Less Macroglia Response After ATX-I Treatment

**FIGURE 8:**
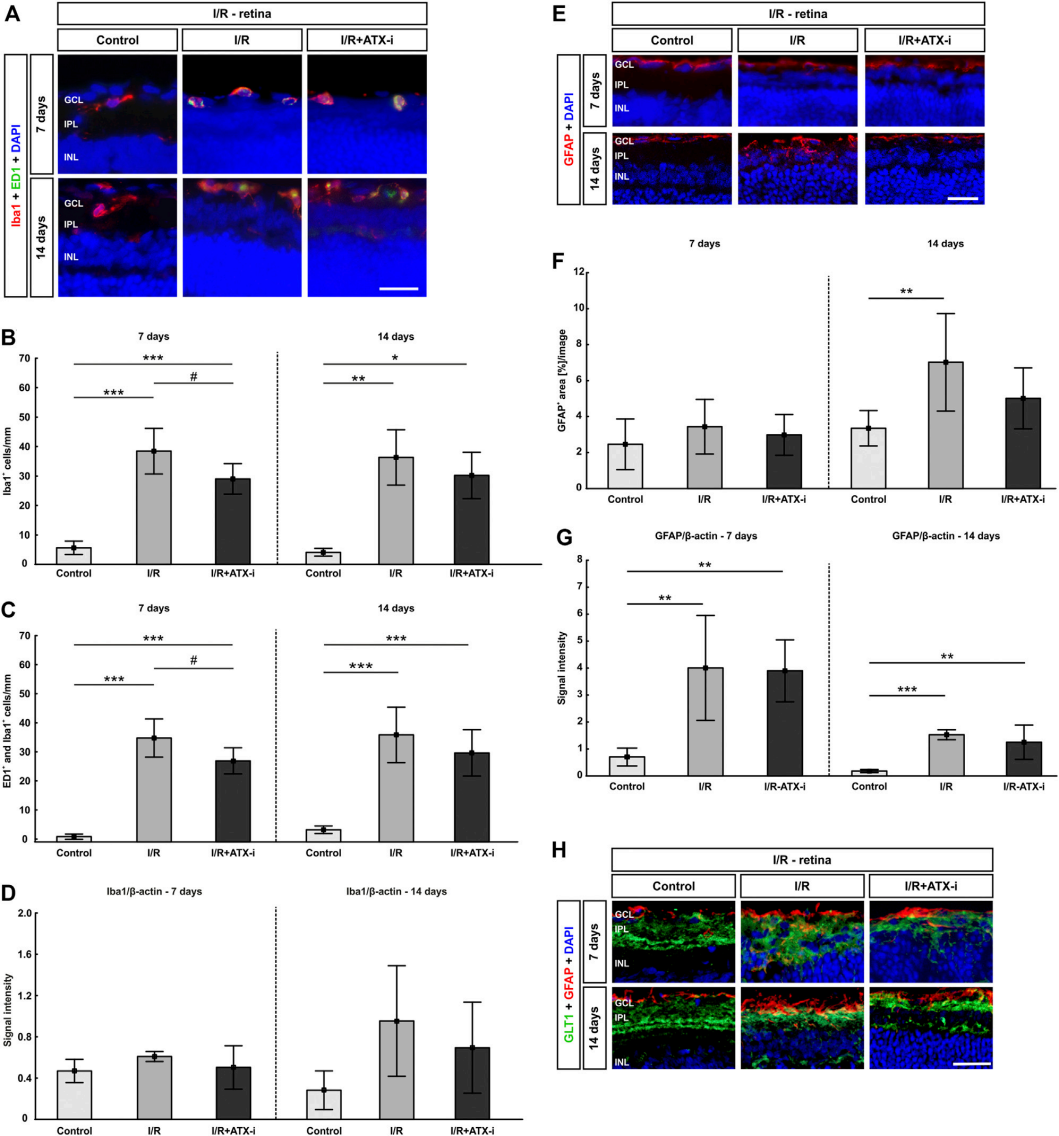
Less retinal microglia after treatment. **(A)** Double immunostaining of microglia with Iba1 (red) and ED1 (green; active ones) after 7 and 14 days. Cell nuclei were marked with DAPI (blue). **(B)** Compared to control retinas, the number of Iba1^+^ microglia was significantly increased in the I/R group and in the I/R + ATX-i group at both points in time. However, 7 days after ischemia induction, significantly fewer Iba1^+^ cells were noted in the I/R + ATX-i in comparison to the I/R group. **(C)** Accordingly, significantly more ED1^+^ and Iba1^+^ co-labeled microglia were detected in the I/R and I/R + ATX-i group 7 as well as 14 days after I/R. Significantly fewer activated microglia were counted in the I/R + ATX-i group when compared to I/R retinas at 7 days. **(D)** After 7 days, no differences were seen in Iba1 protein level in retinal samples of both I/R groups via Western Blot. A trend to higher Iba1 protein levels was noted 14 days after ischemia induction in the I/R group, but not in the I/R + ATX-i group in comparison to control retinas. **(E)** Retinal sections were stained with GFAP (red) to detect astrocytes, while DAPI (blue) counterstained cell nuclei. **(F)** 7 days after ischemia, no changes could be observed in the I/R and I/R + ATX-i group concerning the GFAP^+^ area in comparison to the control group. In contrast, 14 days after ischemia, the GFAP^+^ area was significantly increased in the I/R group, while no changes were detected in the I/R + ATX-i group. **(G)** GFAP analysis via Western Blot showed a significant increase in GFAP signal intensity in both I/R groups after 7 and 14 days. **(H**) Retinas were co-labelled with antibodies against GFAP (red) and GLT1 (green). DAPI visualized cell nuclei (blue). In control retinas, GLT1 staining was mostly observed in the inner plexiform layer. A stronger expression of GLT1 and more co-localization with GFAP seemed to appear in the I/R groups, especially at 7 days. In I/R + ATX-i retinas, a co-localization with GFAP was not that prominent anymore. Values are mean ± SD. 7 days: 5–6 retinas/group; 14 days: 6–7 retinas/group. Abbreviations: GCL = ganglion cell layer; IPL = inner plexiform layer; INL = inner nuclear layer. Scale bars = 20 μm *p < 0.05; **p < 0.01; ***p < 0.001; ^#^ *p* < 0.05.

The I/R (38.44 ± 7.75 cells/mm; *p* < 0.001) and I/R + ATX-i group (29.04 ± 5.19 cells/mm; *p* < 0.001) displayed significantly more Iba1^+^ microglia 7 days after ischemia (control: 5.65 ± 2.29 cells/mm). However, the number of Iba1^+^ cells was significantly lower in I/R + ATX-i retinas compared to I/R ones (*p* = 0.045; Figures 8A,B). After 14 days, induction of ischemia led to a significant increase of Iba1^+^ microglia cells in the I/R (45.5 ± 11.78 cells/mm; *p* = 0.003) and I/R + ATX-i group (41.17 ± 9.33 cells/mm; *p* = 0.013), when compared to the control retinas (26.28 ± 3.33 cells/mm; Figures 8A,B).

A significant increase in ED1^+^ and Iba1^+^ co-labeled cells was seen in the I/R (34.74 ± 6.57 cells/mm; *p* < 0.001) as well as the I/R + ATX-i group (26.91 ± 4.48 cells/mm; *p* < 0.001) in comparison to controls (0.81 ± 0.91 cells/mm) 7 days after ischemia induction. However, a significant decrease in the number of activated microglia was observed in the I/R + ATX-i group when compared to the I/R group (*p* = 0.045; Figures 8A,C). At 14 days, significantly more activated microglia were counted in the I/R (34.20 ± 9.12 cells/mm; *p* < 0.001) and the I/R + ATX-i group (28.27 ± 7.63 cells/mm; *p* < 0.001) in contrast to control retinas (2.99 ± 1.26 cells/mm; Figures 8A,C).

In regard to Iba1 proteins levels, there were no differences between the control group (0.47 ± 0.11) and ischemic retinas (0.61 ± 0.05; *p* = 0.292) as well as I/R + ATX-i treated eyes (0.50 ± 0.21; *p* = 0.921; Figure 8D) at 7 days after ischemia via Western Blot analysis. 14 days after I/R, there was a trend to elevated Iba1 protein levels in the I/R group (0.95 ± 0.54; *p* = 0.064) was noted, but not in the I/R + ATX-i group (0.69 ± 0.44; *p* = 0.309) when compared to control retinas (0.28 ± 0.19; Figure 8D).

7 days after I/R, the GFAP^+^ area in ischemic retinas was slightly increased (3.44 ± 1.52 area [%]/section; *p* = 0.456), whereas the area in the I/R + ATX-i group (2.98 ± 1.13 area [%]/section) was similar to the control group (2.46 ± 1.41 area [%]/section; *p* = 0.823; Figures 8E,F). After 14 days, the GFAP^+^ area was significantly increased in ischemic eyes (7.02 ± 2.71 area [%]/section; *p* = 0.010), while no differences were revealed in the I/R + ATX-i group (5.01 ± 1.70 area [%]/section; *p* = 0.251) compared to the control group (3.35 ± 0.98 area [%]/section; Figures 8E,F).

At 7 days, the GFAP protein level, detected via Western Blot, was significantly upregulated in the I/R (4.00 ± 1.95; *p* = 0.005) and in the I/R + ATX-i group (3.90 ± 1.14; *p* = 0.006) compared to controls (0.71 ± 0.33; Figure 8G). Concerning the GFAP protein level at 14 days, it was significantly upregulated in the I/R group (1.60 ± 0.19; *p* < 0.001). In accordance with the immunohistological data, the protein level in the I/R + ATX-i group (1.31 ± 0.66) appeared to be lower than in the I/R group (*p* = 0.470), while still significantly increased in comparison to controls (0.19 ± 0.06; *p* = 0.002; Figure 8G).

In addition, retinas were co-labeled with anti-GFAP and anti-GLT1 7 and 14 days after I/R. In control retinas, GLT1 staining was predominantly observed in the inner plexiform layer at both points in time. A stronger expression of GLT1 and more co-localization with GFAP seemed to appear in the I/R groups especially at 7 days. In I/R + ATX-i retinas, a co-localization with GFAP was not that prominent anymore (Figure 8H).

##### Only Light Protection of Optic Nerve Neurofilaments With ATX-I

**FIGURE 9:**
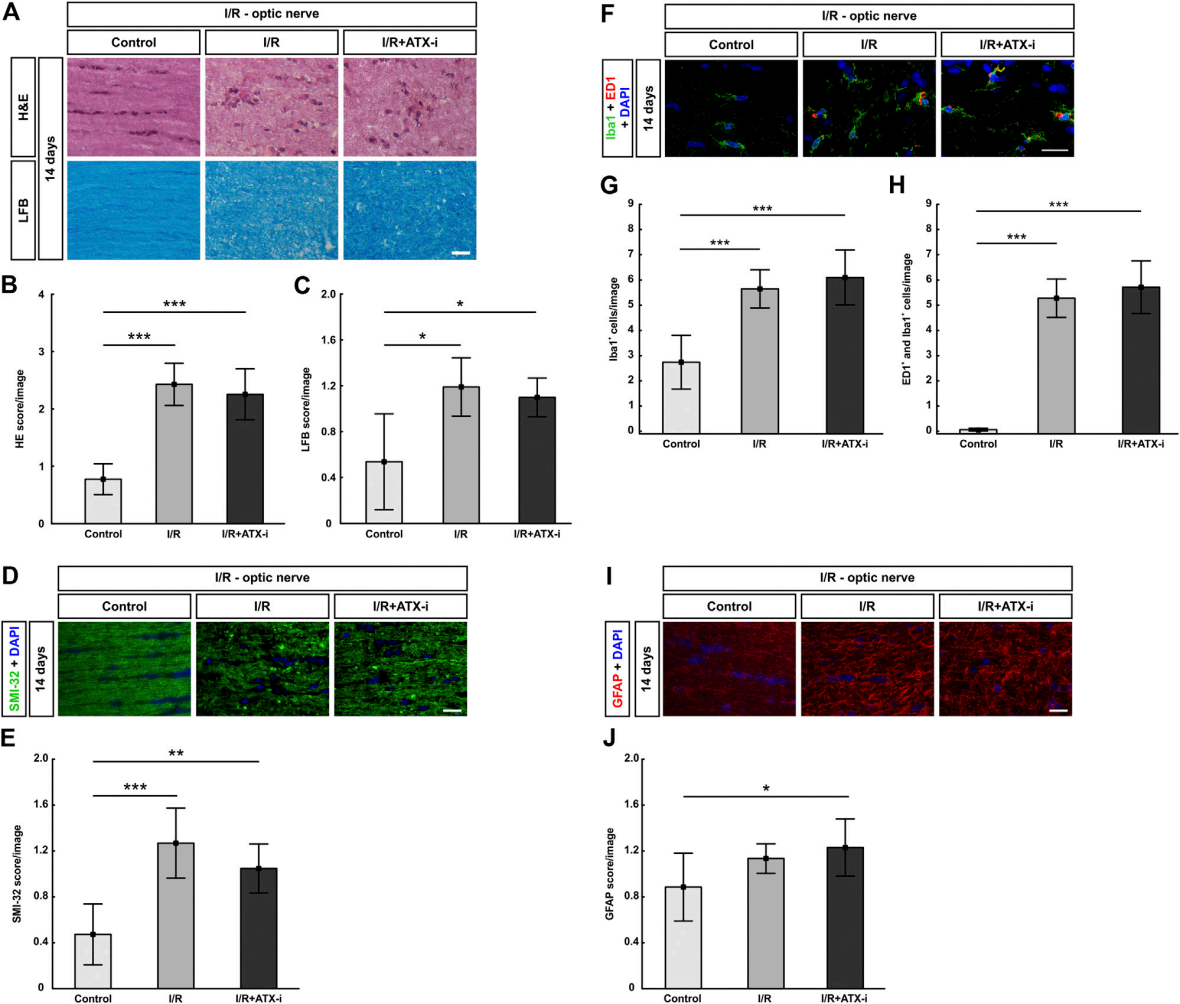
No improvement of I/R optic nerves. **(A)** 14 days after I/R induction, staining with H&E was performed to visualize the optic nerve morphology and possible cell infiltration, while LFB was used for detection of demyelination signs. **(B)** Significantly more inflammatory cells (higher H&E score) were observed in the optic nerves of the I/R and the I/R + ATX-i group. **(C)** A significant loss of myelin (higher LFB score) was noticed in I/R and I/R + ATX-i optic nerves compared to control ones. **(D)** The longitudinal optic nerve sections were labelled with SMI-32 (green) and DAPI (blue; cell nuclei) to detect possible neurofilament changes. **(E)** A significant structural distortion was revealed of the optic nerves of the I/R and I/R + ATX-i group. **(F)** Double immunostaining of microglia with Iba1 (green) and ED1 (red) 14 days post I/R, cell nuclei were marked with DAPI (blue). **(G)** In comparison to controls, the number of Iba1^+^ microglia was significantly increased in the I/R and in the I/R + ATX-i group. **(H)** In accordance, significant more ED1^+^ and Iba1^+^ co-labeled cells were detected in the optic nerves of both ischemic groups, with and without ATX-i treatment. **(I)** Anti-GFAP (red) was used to determine possible alterations in astrocyte expression, while DAPI (blue) counterstained cell nuclei. **(J)** Scoring of the GFAP-labelled tissue showed a significant higher score in the I/R + ATX-i group in comparison to control optic nerves. Values are mean ± SD. 5–6 nerves/group. Scale bars = 20 μm *p < 0.05; **p < 0.01; ***p < 0.001.

14 days after I/R, both the I/R (2.43 ± 0.37; *p* < 0.001) and the I/R + ATX-I (2.25 ± 0.44; *p* < 0.001) group displayed a significantly higher H&E score in the optic nerves compared to the control group (0.77 ± 0.27; Figures 9A,B).

Regarding the LFB score, the I/R (1.19 ± 0.25; *p* = 0.011) as well as the I/R + ATX-i group (1.09 ± 0.17, *p* = 0.016) showed a higher score and bright areas in the optic nerves indicating significant demyelination compared to control nerves (0.54 ± 0.42; Figures 9A,C).

Aligned neurofilament fibers were found in the control group, while a significant structural distortion was detected by SMI-32 scoring in the optic nerves of the I/R (1.27 ± 0.31; *p* < 0.001) and the I/R + ATX-i group (I/R + ATX-i: 1.05 ± 0.21; control: 0.47 ± 0.27, *p* = 0.002; Figures 9D,E). The I/R + ATX-i optic nerves, however, appeared to be slightly less damaged at the 14-days time point (Figures 9D,E).

Regarding microglia, the number of Iba1^+^ cells was significantly upregulated in the I/R (5.65 ± 0.75 cells/image; *p* < 0.001) and I/R + ATX-i (6.10 ± 1.09 cells/image; *p* < 0.001) group in comparison to control optic nerves (2.74 ± 1.06 cells/image; Figures 9F,G). Likewise, the number of activated microglia (ED1^+^ and Iba1^+^) was significantly increased in I/R (5.28 ± 0.76 cells/image; *p* < 0.001) and I/R + ATX-i optic nerves (5.71 ± 1.04 cells/image; *p* < 0.001) in comparison to controls (0.06 ± 0.06 cells/image; Figures 9F,H).

GFAP scoring, for a possible astrogliosis, revealed a significant difference between the control (0.89 ± 0.30) and the I/R + ATX-i group (1.23 ± 0.25) at 14 days (*p* = 0.039). A slight trend to a higher score in the I/R group (1.13 ± 0.13; *p* = 0.200) compared to controls was also noted (Figures 9I,J).

## 4 Discussion

### 4.1 Medicinal Chemistry

Our initial attempt to find new proprietary ATX-inhibitors was based on the seed compound PF-8380 (**1**) and relied on modifications of the molecule which are in essence: 1) the ZBG, 2) the core region, and 3) the structural element that fits into the lipophilic pocket (Figure 1A). Finally, the benzotriazole moiety was found to be one of the preferred ZBGs because it addressed key issues identified in **1**, such as formation of reactive metabolites while ensuring good potency and properties. The core could be replaced by a number of bicyclic or spirocyclic building blocks that resulted in retention of the *in vitro* potency and improvement of chemical stability over **1**. In combination with the previous modifications, introduction of a trifluoromethoxybenzyl moiety as the lipophilic pocket binder resulting in compound **13** provided the lead compound with the most balanced overall profile with regard to potency (IC_50_ against ATX: 6 nM), lipophilicity (LogD), permeability, solubility, and low microsomal clearance *in vitro*. While for a number of compounds such as **14**, **7**, and **15** the low microsomal clearance *in vitro* did not translate into sufficient exposure in rat after oral dosing for unclear reasons, **13** provided an acceptable PK profile with good oral bioavailability and sufficient half live to allow for subsequent acute and chronic *in vivo* PD studies. In addition, screening of compound **13** in a panel of approximately 70 assays covering a variety of enzymes, receptors, and transporters revealed a low risk for off target effects (Supplementary Table S2).

### 4.2 Evaluation of PK/PD of Compound 13

Sustained ATX inhibition was demonstrated after oral dosing of **13** in an acute PK/PD study in rats. *In vivo*, LPA levels of total LPA or the different LPA species analyzed were lowered in a dose-dependent fashion and ATX activity was also inhibited *ex vivo* when plasma ATX activity was monitored with incubation of the sample (Figure 2). Besides the single dose PK, we evaluated food admix as an alternative route of drug administration which would substantially minimize the stress during chronic drug treatments to the animals compared to gavage or injections.

Based on the PK/PD relationship observed in the acute experiment, administration via food admix of **13** at a dose of 20 mg/kg results in an estimated average ATX inhibition of > 75% during the 2-weeks study at steady state. Therefore, food admix was chosen as the preferred route of administration of **13** for the *in vivo* studies.

### 4.3 *In vivo* Studies

Glaucomatous neuropathy is one of the most common causes of blindness worldwide (Weinreb et al., 2014; Egs, 2017). However, until now, the knowledge about underlying pathomechanisms remains sparse. Besides an elevated IOP, other mechanisms, such as ischemic processes and immunological alterations contribute to disease development (Grus et al., 2008; Joachim et al., 2012a; Schmid et al., 2014; Palmhof et al., 2019). Nonetheless, to date, IOP lowering is the only treatment option (Egs, 2017). Current medical or surgical approaches cannot reverse damage, but only slow down disease progression. Hence, novel, alternative therapies are needed that might eventually stop disease progression.

Based on the compelling link of an overactive ocular ATX-LPA axis with POAG, we sought to investigate if ATX inhibition using the novel ATX-inhibitor (ATX-i (**13**)) might protect RGCs beyond IOP control in two different glaucoma models, namely the EAG and the I/R model. In EAG animals, glaucomatous damage occurs after immunization with ocular antigens independently of IOP (Laspas et al., 2011; Noristani et al., 2016). In the current study, we observed a mild preservation of RGCs in ATX-i (**13**)-treated animals compared to ONA immunized rats (Figures 3C,D). RGCs are the neurons that transmit visual stimuli from the retina to the brain (Mead and Tomarev, 2016) and therefore, protection of these cells will preserve vision. Neither ONA immunization nor treatment with ATX-i (**13**) affected the IOP (Supplementary Figure S1). As previously shown, IOP stayed within the normal range in the EAG model (Laspas et al., 2011; Joachim et al., 2013). Other studies demonstrated that topically or intracamerally administrated ATX-inhibitors acutely decreased the IOP in healthy rabbits as well as in mice with elevated pressure due to laser peripheral iridotomy (Iyer et al., 2012; Nagano et al., 2019). In contrast, ATX-i (**13**), which was administered via the oral route, did not lower IOP in both models. This suggests that, as shown in mice (Nagano et al., 2019), IOP might have to be experimentally increased, in order to demonstrate IOP lowering by ATX inhibition in rodent models.

We noted an increase in serum and aqueous humor IgG levels in ONA immunized animals, while the IgG levels were lower in rats treated with the ATX-i (**13**) (Figures 3A,B). In prior studies, IgG deposits have been observed in POAG patients (Gramlich et al., 2013; Von Thun Und Hohenstein-Blaul et al., 2017) as well as in EAG retinas, optic nerves, and aqueous humor (Laspas et al., 2011; Joachim et al., 2013). ATX-i (**13**) lowered elevated IgG levels preferably in the aqueous humor and therefore contributes to a diminished immune response in the eye. In addition, as a consequence of increased IgG levels, significantly more microglia and activated microglia cells were observed in ONA animals (Figures 4A–C). The reduced level of inflammation was also reflected in lower numbers of total and activated microglia in the ATX-i (**13**)-treated group. Microglia seem to play an important role in glaucoma in general (Karlstetter et al., 2015) which is in line with an activation of microglia observed in the EAG model previously (Joachim et al., 2012a; Noristani et al., 2016; Reinehr et al., 2019b). Microglia are responsive to LPA via LPA receptors, which regulate cell morphology, migration, and growth factor production (Schilling et al., 2004; Fujita et al., 2008; Muessel et al., 2013). In a septic mouse model, the blockade of LPA1 receptor signaling diminished microglia activation, transformation, and proliferation (Kwon et al., 2018). Furthermore, the LPA1 receptor was also shown to contribute to demyelination in spinal cord injury through microglia activation (Santos-Nogueira et al., 2015).

Besides microglia, we also investigated the macroglia response. Treatment with ATX-i (**13**) led to a decreased GFAP^+^ area in contrast to ONA animals (Figures 4D–F), which is in line with the observed LPA-mediated proliferation of astrocytes *in vitro* (Shano et al., 2008). In LPA-primed astrocytes, several factors where secreted, which were responsible for neuronal differentiation, axon growth, as well as epidermal growth factor signaling (Tabuchi et al., 2000; Spohr et al., 2011). This suggests that inhibition of ATX lowers the observed GFAP response in the EAG model due to decreased LPA levels.

In a previous study, ONA led to nerve fiber and neurofilament degeneration 28 days after immunization (Noristani et al., 2016). In this study, however, we only detected an early sign of optic nerve degeneration such as disruption of the neurofilament, which could be prevented by ATX-i (**13**) treatment (Figures 5D,E). Interestingly, a higher number of microglia cells were observed in ONA and ONA + ATX-i optic nerves in contrast to controls. However, only few activated microglia were detected in all groups. In summary, ATX-i (**13**) did not lead to notable effects on microglia with the relative mild phenotype observed in this study (Figures 5F–H).

In contrast to the EAG model, I/R leads to a severe and early retinal damage with RGC loss occurring already 2 h after ischemia induction. Cone bipolar cells as well as cone photoreceptors decline 6 and 12 h after ischemia (Palmhof et al., 2019). In the study presented here, we analyzed the effect of ATX-i (**13**) at days 7 and 14 after I/R. Analyses via ERG revealed no significant protective effects of ATX-i (**13**) at day 7 on the I/R-induced loss of retinal function. Interestingly, at day 14, the a-wave amplitude, reflecting the photoreceptor response, was preserved in animals treated with ATX-i (**13**) (Figures 6A–D).

I/R injury affects the whole retina, resulting in a retinal thinning (Joachim et al., 2017; Palmhof et al., 2019). In this study, ATX-i (**13**) did not preserve GCL thickness at day 7 but to some extent at day 14 (Figures 6E,F). To investigate the retinal cell types, immunohistology and Western Blot analyses were performed. Similar to the EAG model, ATX-i (**13**) could also mildly protect RGCs (Figures 7A–C), although I/R in general leads to more severe retinal damage (Joachim et al., 2017). No effect of ATX-i (**13**) was noted in the number of cleaved caspase 3^+^ apoptotic cells. At both points in time, more apoptotic cells in the GCL were noted in I/R and I/R + ATX-i retinas (Figures 7D,E). Previously, more cleaved caspase 3^+^ cells were observed already 2 h after I/R induction (Wagner et al., 2020). The severe damage following the ischemia induction could explain the lack of treatment effects on apoptotic cell numbers.

Ischemia injury has been shown to involve activation of microglia cells (Zhang et al., 2005; Cho et al., 2011; Schmid et al., 2014; Wagner et al., 2020), which play an important role as phagocytic cells to remove cell debris (Xu et al., 2016). In line with this, the total number as well as the number of activated microglia was elevated after I/R at both points in time. ATX-i (**13**) was able to reduce the retinal microglia number at 7, but not at 14 days, suggesting increased ATX-i (**13**) activity soon after I/R (Figures 8A–D). It is possible that the damage of the retina developed further independent of ATX-mediated LPA formation after day 7, which resulted in higher microglia counts, even in the treatment group at day 14.

LPA-dependent microglia activation was shown to contribute to secondary damage after spinal cord injury involving activation of LPA1 receptors. In this model, LPA levels increased in the spinal cord parenchyma during the first 14 days (Santos-Nogueira et al., 2015). Similarly, LPA1 receptor-mediated microglia activation was noted in a mouse model for transient middle cerebral artery occlusion (Gaire et al., 2019). It seems that elevated LPA levels can contribute to severe neuronal tissue damage by increased activation of microglia over time.

In contrast to the EAG model, a significant increase of GFAP^+^ area was notable in I/R retinas after 14 days (Figures 8E–H). ATX-i (**13**) and the resulting LPA lowering seemed to ameliorate this long-lasting proliferation of astrocytes after ischemia.

Similar to the EAG model, inhibition of ATX had no effect on the neurodegeneration of the optic nerves 7 and 14 days after I/R (Figure 9). Since ATX-i and consequently LPA lowering did not seem to be sufficient for rescuing the optic nerve structures it is suggested that major factors other than elevated LPA levels and activated microglia contribute to the damaging effects.

## 5 Conclusion

We describe the generation of a set of novel spirocyclic and bicyclic ATX-inhibitors. The lead compound (**13**, ATX-i) of this new series with improved properties was suitable for investigation in two glaucoma *in vivo* models after oral administration in both an acute and chronic setting. Dose-dependent LPA lowering in rat was confirmed after acute administration. In the EAG and the I/R model, ATX inhibition led to some RGC protection and less gliosis, especially in the retina. Reduced microglia numbers and activation upon ATX-i (**13**) treatment suggests that neuroprotective effects are the result of LPA lowering and of reduced LPA receptor activity. Since ATX-i (**13**) was not able to fully protect cells from severe ischemic retinal and optic nerve damage, it is suggested that major factors other than elevated LPA levels and activated microglia contribute to the damaging effects in both models. In summary, compound ATX-i (**13**) proved to be suitable to identify a crucial role for an overactive ATX-LPA axis in glaucomatous retinal damage. In addition to the demonstrated ATX-i mediated IOP lowering, retinal ATX-i might further contribute to reduce disease progression.

## Data Availability

The raw data supporting the conclusions of this article will be made available by the authors, without undue reservation.
