# Supplementary material for: Synthesis, Characterization, and in vivo Evaluation of a Novel Potent Autotaxin-Inhibitor
Source: Front Pharmacol. 2022 Jan 18;12:699535. doi: 10.3389/fphar.2021.699535 (PMC8807399; doi:10.3389/fphar.2021.699535)
Supplement: Supplementary file 1 [file DataSheet1.docx]

Supplementary Material

# Supplementary Data

## Synthetic procedures and analytical data for compounds 2 - 6 and 8 - 15

S1.1: *trans*-3,5-Dichlorobenzyl 2-(2-oxo-2,3-dihydrobenzo[d]oxazole-6-carbonyl)hexa-hydro-*1H*-pyrrolo[3,4-c]pyridine-5(*6H*)-carboxylate (**2**)

Step 1: *trans*-2-tert-Butyl 5-(3,5-dichlorobenzyl) tetrahydro-*1H*-pyrrolo[3,4-c]pyridine-2,5(*3H,6H*)-dicarboxylate (**18**)

To a solution of (3,5-dichlorophenyl)methanol (**16**; 403 mg, 2.21 mmol) in dichloromethane (10 mL) was added *N,N'*-carbonyldiimidazole (388 mg, 2.32 mmol) at RT, then after 3 h at RT *trans*-octahydro-pyrrolo[3,4-c]pyridine-2-carboxylic acid tert-butyl ester (**17**; CAS-RN 1251014-37-5; 500 mg, 2.21 mmol) and triethylamine (224 mg, 2.21 mmol) were added and the reaction was stirred for another 18 h. The reaction mixture was partitioned between ethyl acetate and water. The organic layer was washed with brine, dried over magnesium sulfate, filtered, and evaporated. Chromatography (silica gel; heptane-ethyl acetate gradient) produced the title compound **18** (825 mg, 87%) as a colorless gum, MS: 373.5 [M+H–isobutene]^+^.

^1^H NMR (CDCl_3_, 300 MHz) δ 7.3-7.35 (m, 1H), 7.2-7.25 (m, 2H), 5.07 (br d, 2H, J=7.5 Hz), 4.36 (br s, 2H), 3.5-3.7 (m, 2H), 2.88 (br t, 3H, J=10.4 Hz), 2.62 (br s, 1H), 1.89 (br t, 1H, J=11.5 Hz), 1.6-1.7 (m, 2H), 1.46 (s, 9H), 1.35 (br d, 1H, J=13.7 Hz).

Step 2: *trans*-3,5-Dichlorobenzyl hexahydro-*1H*-pyrrolo[3,4-c]pyridine-5(*6H*)-carboxylate hydrochloride (**19**)

*trans*-2-tert-Butyl 5-(3,5-dichlorobenzyl) tetrahydro-*1H*-pyrrolo[3,4-c]pyridine-2,5(*3H,6H*)-dicarboxylate (**18**; 817 mg, 1.9 mmol) was combined with hydrochloric acid (5–6 M in 2-propanol, 6.9 mL, 34.5 mmol) and stirred at room temperature for 16 h, then the reaction mixture was directly evaporated und the residue was precipitated from ethyl acetate to produce the title compound **19** (576 mg, 83%) as a white solid, MS: 329.4 [M+H]^+^.

^1^H NMR ((CD_3_)_2_SO, 300 MHz) δ 9.17 (br s, 2H), 7.58 (t, 1H, J=1.9 Hz), 7.43 (d, 2H, J=2.0 Hz), 5.09 (br s, 2H), 4.31 (dd, 1H, J=3.0, 12.5 Hz), 4.16 (br d, 1H, J=13.3 Hz), 3.3-3.4 (m, 2H), 2.5-2.9 (m, 4H), 1.88 (br dd, 1H, J=2.1, 12.6 Hz), 1.5-1.7 (m, 2H), 1.26 (dq, 1H, J=4.4, 12.1 Hz).

Step 3: *trans*-3,5-Dichlorobenzyl 2-(2-oxo-2,3-dihydrobenzo[d]oxazole-6-carbonyl)-hexahydro-*1H*-pyrrolo[3,4-c]pyridine-5(*6H*)-carboxylate (**2**)

To a solution of *trans*-3,5-dichlorobenzyl hexahydro-*1H*-pyrrolo[3,4-c]pyridine-5(*6H*)-carboxylate hydrochloride (**19**; 50 mg, 137 µmol) in *N,N*-dimethylformamide (1 mL) were added 4-methylmorpholine (69.1 mg, 684 µmol), 4-amino-3-hydroxybenzoic acid (20.9 mg, 137 µmol), and *O*-(7-azabenzotriazol-1-yl)-*N,N,N’,N’*-tetramethyluronium hexafluoro-phosphate (62.4 mg, 164 µmol) at room temperature. Then after 18 h, 1,1'-carbonyldiimidazole (50.3 mg, 301 µmol) was added. After 1 h, the reaction mixture was partitioned between ethyl acetate and 1 M aq. hydrochloric acid solution. The organic layer was washed with brine, dried over magnesium sulfate, filtered, and evaporated. Chromatography (silica gel, gradient dichloromethane dichloromethane/methanol/25% aq. ammonia solution 90:10:0.25) produced the title compound **2** (35 mg, 52%) as a light-yellow gum.

^1^H NMR (CDCl_3_, 300 MHz) δ 9.17 (br s, 1H), 7.2-7.4 (m, 5H), 7.04 (d, 1H, J=7.9 Hz), 5.08 (br s, 2H), 4.3-4.6 (m, 2H), 3.8-4.0 (m, 1H), 3.5-3.7 (m, 1H), 3.1-3.4 (m, 2H), 2.5-2.9 (m, 2H), 1.7-1.9 (m, 2H), 1.6-1.7 (m, 1H), 1.3-1.5 (m, 1H).

LC-HRMS (m/z): [M+H]^+^ calcd for [C_23_H_21_Cl_2_N_3_O_5_+H]^+^: 490.0931, found: 490.0934, UV purity (230-300 nm): 98%.

S1.2: *trans*-3,5-Dichlorobenzyl 7-(2-oxo-2,3-dihydrobenzo[d]oxazole-6-carbonyl)-2,7-diaza-spiro[3.5]nonane-2-carboxylate (**3**)

The title compound **3** was produced in analogy to compound **2**, replacing *trans*-octahydro-pyrrolo[3,4-c]pyridine-2-carboxylic acid tert-butyl ester in step 1 by 2,7-diazaspiro[3.5]nonane-7-carboxylic acid tert-butyl ester (CAS-RN 896464-16-7). Compound **3** was isolated as a light-yellow gum.

^1^H NMR (CDCl_3_, 300 MHz) δ 8.87 (br s, 1H), 7.2-7.3 (m, 5H), 7.05 (d, 1H, J=7.9 Hz), 5.04 (s, 2H), 3.80 (s, 4H), 3.3-3.7 (m, 4H), 1.7-1.9 (m, 4H).

LC-HRMS (m/z): [M+H]^+^ calcd for [C_23_H_21_Cl_2_N_3_O_5_+H]^+^: 490.0931, found: 490.0936, UV purity (230-300 nm): 99%.

S1.3: 3,5-Dichlorobenzyl 2-(2-oxo-2,3-dihydrobenzo[d]oxazole-6-carbonyl)-2,7-diaza-spiro[3.5]-nonane-7-carboxylate (**4**)

The title compound **4** was produced in analogy to compound **2**, replacing *trans*-octahydro-pyrrolo[3,4-c]pyridine-2-carboxylic acid tert-butyl ester in step 1 by 2,7-diazaspiro[3.5]nonane-2-carboxylic acid tert-butyl ester (CAS-RN 236406-55-6). Compound **4** was isolated as a light-yellow solid.

^1^H NMR ((CD_3_)_2_SO, 300 MHz) δ 11.90 (br s, 1H), 7.4-7.6 (m, 5H), 7.13 (d, 1H, J=8.1 Hz), 5.06 (s, 2H), 4.0-4.1 (m, 2H), 3.77 (br s, 2H), 3.3-3.5 (m, 4 H), 1.6-1.7 (m, 4H).

LC-HRMS (m/z): [M+H]^+^ calcd for [C_23_H_21_Cl_2_N_3_O_5_+H]^+^: 490.0931, found: 490.0935, UV purity (230-300 nm): 100%.

S1.4: (*3aR,6aS*)-3,5-Dichlorobenzyl 5-(2-oxo-2,3-dihydrobenzo[d]oxazole-6-carbonyl)-hexahydropyrrolo[3,4-c]pyrrole-2(*1H*)-carboxylate (**5**)

The title compound was **5** produced in analogy to compound **2**, replacing *trans*-octahydro-pyrrolo[3,4-c]pyridine-2-carboxylic acid tert-butyl ester in step 1 by (*3aR,6aS*)-hexahydro-pyrrolo[3,4-c]pyrrole-2(*1H*)-carboxylic acid, tert-butyl ester (CAS-RN 250275-15-1). Compound **5** was isolated as a light-yellow foam.

^1^H NMR ((CD_3_)_2_SO, 300 MHz) δ 11.85 (br s, 1H), 7.56 (br s, 1H), 7.49 (d, J = 1.5, 1H), 7.44 (br s, 2H), 7.35 (dd, 1H, J=1.5, 8.1 Hz), 7.11 (d, 1H, J=8.1 Hz), 5.07 (s, 2H), 3.1-3.9 (m, 8H), 2.8-3.0 (m, 2H).

LC-HRMS (m/z): [M+H]^+^ calcd for [C_22_H_19_Cl_2_N_3_O_5_+H]^+^: 476.0775, found: 476.0778, UV purity (230-300 nm): 93%.

S1.5: *trans*-2-(*1H*-Benzotriazole-5-carbonyl)-octahydro-pyrrolo[3,4-c]pyridine-5-carboxylic acid 3,5-dichloro-benzyl ester (**6**)

*O*-(7-Azabenzotriazol-1-yl)-*N,N,N’,N’*-tetramethyluronium hexafluoro-phosphate (260 mg, 684 µmol) was added at 0°C to a mixture of *trans*-3,5-dichlorobenzyl hexahydro-*1H*-pyrrolo[3,4-c]pyridine-5(*6H*)-carboxylate hydrochloride (**19**; 250 mg, 684 µmol), *N*-methylmorpholine (346 mg, 3.42 mmol), and *1H*-benzo[d][1,2,3]triazole-5-carboxylic acid (112 mg, 684 µmol) in *N,N*-dimethylformamide (7 mL). The ice bath was removed, then after 16 h the reaction mixture was partitioned between ethyl acetate and sat. aq. ammonium chloride solution. The organic layer was washed with brine, dried over magnesium sulfate, filtered, and evaporated. Chromatography (silica gel, gradient dichloromethane to dichloromethane/methanol/25% aq. ammonia solution 90:10:0.25) produced the title compound **6** (325 mg, 100%) as a light-yellow foam.

^1^H NMR ((CD_3_)_2_SO, 300 MHz) δ 15.89 (br s, 1H), 8.09 (br s, 1H), 7.93 (br d, 1H, J=8.7 Hz), 7.5-7.6 (m, 2H), 7.4-7.5 (m, 2H), 5.0-5.2 (m, 2H), 4.1-4.4 (m, 2H), 3.6-3.8 (m, 1H), 3.4-3.6 (m, 1H), 3.2-3.3 (m, 1H), 3.0-3.2 (m, 1H), 2.6-2.8 (m, 1H), 2.5-2.6 (m, 1H), 1.9-2.0 (m, 1H), 1.6-1.9 (m, 2H), 1.1-1.4 (m, 1H).

LC-HRMS (m/z): [M+H]^+^ calcd for [C_22_H_21_Cl_2_N_5_O_3_+H]^+^: 474.1094, found: 474.1102, UV purity (230-300 nm): 99%.

S1.6: (*3aR,6aS*)-3,5-dichlorobenzyl 5-(*1H*-benzo[d][1,2,3]triazole-5-carbonyl)hexahydro-pyrrolo[3,4-c]pyrrole-2(*1H*)-carboxylate (**8**)

The title compound **8** was produced in analogy to compound **6**, replacing *trans*-octahydro-pyrrolo[3,4-c]pyridine-2-carboxylic acid tert-butyl ester by (*3aR,6aS*)-hexahydro-pyrrolo[3,4-c]pyrrole-2(*1H*)-carboxylic acid, tert-butyl ester (CAS-RN 250275-15-1). Compound **6** was isolated as a light-yellow foam.

^1^H NMR (CDCl_3_, 300 MHz) δ 14.89 (br s, 1H), 8.04 (m, 1H), 7.80 (br s, 1H), 7.5-7.7 (m, 1H), 7.2-7.3 (m, 3H), 5.10 (m, 2H), 3.3-4.1 (m, 8H), 2.9-3.2 (m, 2H).

LC-HRMS (m/z): [M+H]^+^ calcd for [C_21_H_19_Cl_2_N_5_O_3_+H]^+^: 460.0938, found: 460.0944, UV purity (230-300 nm): 98%.

S1.7: 3,5-Dichlorobenzyl 2-(*1H*-benzo[d][1,2,3]triazole-5-carbonyl)-2,7-diazaspiro[3.5]-nonane-7-carboxylate (**9**)

The title compound **9** was produced in analogy to compound **6**, replacing *trans*-octahydro-pyrrolo[3,4-c]pyridine-2-carboxylic acid tert-butyl ester by 2,7-diazaspiro[3.5]nonane-2-carboxylic acid tert-butyl ester (CAS-RN 236406-55-6). Compound **9** was isolated as a light-yellow foam.

^1^H NMR (CDCl_3_, 300 MHz) δ 14.65 (br s, 1 H) 8.1-8.3 (m, 1H), 7.7-7.9 (m, 2H), 7.2-7.3 (m, 3H), 5.07 (s, 2H), 4.12 (br s, 2H), 4.05 (br s, 2H), 3.49 (br t, 4H, J=5.4 Hz), 1.83 (br d, 4H, J=5.4 Hz).

LC-HRMS (m/z): [M+H]^+^ calcd for [C_22_H_21_Cl_2_N_5_O_3_+H]^+^: 474.1094, found: 474.1101, UV purity (230-300 nm): 100%.

S1.8: *cis*-3,5-Dichlorobenzyl 2-(*1H*-benzo[d][1,2,3]triazole-5-carbonyl)octahydropyrrolo-[3,4-d]azepine-6(*7H*)-carboxylate (**10**)

The title compound **10** was produced in analogy to compound **6**, replacing *trans*-octahydro-pyrrolo[3,4-c]pyridine-2-carboxylic acid tert-butyl ester by (*3aR,8aS*)-tert-butyl octahydropyrrolo[3,4-d]azepine-2(*1H*)-carboxylate (CAS-RN 1251013-07-6). Compound **10** was isolated as a white foam.

^1^H NMR (CDCl_3_, 300 MHz) δ 14.03 (br s, 1H) 8.04 (br s, 1H), 7.77 (br s, 1H), 7.60 (br s, 1H), 7.2-7.3 (m, 3H), 5.09 (br s, 2H), 3.3-4.0 (m, 8H), 2.4-2.6 (m, 2H), 1.7-2.0 (m, 4H).

LC-HRMS (m/z): [M+H]^+^ calcd for [C_23_H_23_Cl_2_N_5_O_3_+H]^+^: 488.1251, found: 488.1256, UV purity (230-300 nm): 99%.

S1.9: (*E*)-1-((*3aR,6aR*)-5-(*1H*-benzo[d][1,2,3]triazole-5-carbonyl)hexahydropyrrolo[3,4-c]pyrrol-2(*1H*)-yl)-3-(4-(trifluoromethoxy)phenyl)prop-2-en-1-one (**11**)

*O*-(7-Azabenzotriazol-1-yl)-*N,N,N’,N’*-tetramethyluronium hexafluoro-phosphate (253 mg, 666 µmol) was added at 0°C to a suspension of (*1H*-benzo[d][1,2,3]triazol-5-yl)((*3aR,6aR*)-hexahydropyrrolo[3,4-c]pyrrol-2(*1H*)-yl)methanone hydrochloride (**27**, 220 mg, 666 µmol), *N*-methylmorpholine (404 mg, 4.00 mmol), and (*E*)-3-(4-(trifluoromethoxy)phenyl)acrylic acid (159 mg, 666 µmol) in *N,N*-dimethylformamide (20 mL), and the reaction mixture was allowed to reach room temperature over 16 h and was then partitioned between dichloromethane and sat. aq. sodium hydrogen carbonate solution. The organic layer was washed with brine, dried over magnesium sulfate, filtered and evaporated. The residue was triturated in ethyl acetate and the precipitate was collected by filtration and dried *in vacuo* to produce the title compound **11** (224 mg, 71%) as a light-yellow solid.

^1^H NMR ((CD_3_)_2_SO, 300 MHz) δ 15.91 (br s, 1H), 8.12 (br s, 1H), 7.95 (br s, 1H), 7.85 (t, 2H, J=8.8 Hz), 7.62 (br s, 1H), 7.52 (d, 1H, J=15.6 Hz), 7.39 (t, 2H, J=7.1 Hz), 6.99 (dd, 1H, J=15.6, 16.9 Hz), 3.7-4.1 (m, 4H), 3.0-3.6 (m, 4H), 2.2-2.5 (m, 2H).

LC-HRMS (m/z): [M+H]^+^ calcd for [C_23_H_20_F_3_N_5_O_3_+H]^+^: 472.1591, found: 472.1593, UV purity (230-300 nm): 99%.

S1.10: 1-[(*3aS,6aS*)-5-(*1H*-Benzotriazole-5-carbonyl)-hexahydro-pyrrolo[3,4-c]pyrrol-2-yl]-2-(4-trifluoromethoxy-phenoxy)-ethanone (**12**)

*O*-(7-Azabenzotriazol-1-yl)-*N,N,N’,N’*-tetramethyluronium hexafluoro-phosphate (53.1 mg, 140 µmol) was added at 0°C to a suspension of (*1H*-benzo[d][1,2,3]triazol-5-yl)((*3aR,6aR*)-hexahydropyrrolo[3,4-c]pyrrol-2(*1H*)-yl)methanone hydrochloride (**27**; 41 mg, 140 µmol), *N*-methylmorpholine (70.6 mg, 698 µmol), and 2-(4-(trifluoromethoxy)phenoxy)acetic acid (33.0 mg, 140 µmol) in *N,N*-dimethylformamide (5 mL). After 30 min, the ice-bath was removed and then after 16 h the reaction mixture was partitioned between dichloromethane and sat. aq. sodium hydrogen carbonate solution. The organic layer was washed with brine, dried over magnesium sulfate, filtered, and evaporated. The residue was dissolved in dichloromethane (0.5 mL), then after standing for 1 h at room temperature, a solid precipitated. Heptane and tert-butyl methyl ether (1:1) were added to the suspension and the precipitate was collected by filtration and dried *in vacuo* to afford the title compound **12** (48 mg, 72 %) as a white solid.

^1^H NMR ((CD_3_)_2_SO, 300 MHz) δ 15.91 (br s, 1H), 8.19 (br s, 1H), 7.96 (br s, 1H), 7.60 (br s, 1H), 7.28 (dd, 2H, J=1.8, 9.1 Hz), 7.0-7.1 (m, 2H), 4.7-4.9 (m, 2H), 3.5-3.9 (m, 4H), 2.9-3.5 (m, 4H), 2.2-2.5 (m, 2H)

LC-HRMS (m/z): [M+H]^+^ calcd for [C_22_H_20_F_3_N_5_O_4_+H]^+^: 476.1540, found: 476.1542, UV purity (230-300 nm): 100%.

S1.11: (*3aS,6aS*)-5-(*1H*-Benzotriazole-5-carbonyl)-hexahydro-pyrrolo[3,4-c]pyrrole-2-carboxylic acid 4-trifluoromethoxy-benzyl ester (**13**)

To a solution of (4-(trifluoromethoxy)phenyl)methanol (1.35 g, 6.81 mmol) in acetonitrile (100 mL) was added *N,N'*-carbonyldiimidazole (1.16 g, 7.15 mmol), then after 2.5 h, triethylamine (3.44 g, 34.0 mmol) and (*1H*-benzo[d][1,2,3]triazol-5-yl)((*3aR,6aR*)-hexahydropyrrolo[3,4-c]pyrrol-2(*1H*)-yl)methanone hydrochloride (**27**; 2 g, 6.81 mmol) were added and the reaction mixture was heated to reflux. After 16 h, the reaction mixture was evaporated *in vacuo* and the residue was partitioned between and diluted with ethyl acetate / 2-methyltetrahydrofuran 8:2 and aq. sat. ammonium chloride solution. The organic layer was washed with brine, dried over magnesium sulfate, filtered, and evaporated. The residue was triturated with ethyl acetate and tert-butyl methyl ether. The precipitate was collected by filtration and dried *in vacuo* to afford the title compound **13** (2.15 g, 66%) as a light-brown solid.

^1^H NMR (CDCl_3_, 300 MHz) δ 12.50 (br s, 1H), 8.02 (s, 1H), 7.82 (br d, 1H, J=8.3 Hz), 7.59 (d, 1H, J=8.5 Hz), 7.4-7.4 (m, 2H), 7.1-7.2 (m, 2H), 5.15 (d, 2H, J=2.6 Hz), 3.8-4.1 (m, 2H), 3.6-3.7 (m, 2H), 3.3-3.5 (m, 2H), 3.1-3.2 (m, 2H), 2.2-2.5 (m, 2H).

LC-HRMS (m/z): [M+H]^+^ calcd for [C_22_H_20_F_3_N_5_O_4_+H]^+^: 476.1540, found: 476.1545, UV purity (230-300 nm): 99%.

S1.12: 1-[(*3aR,6aR*)-5-(*1H*-benzotriazole-5-carbonyl)-hexahydro-pyrrolo[3,4-c]pyrrol-2-yl]-3-(4-trifluoromethoxy-phenyl)-propan-1-one (**14**)

*O*-(7-Azabenzotriazol-1-yl)-*N,N,N’,N’*-tetramethyluronium hexafluoro-phosphate ((51.8 mg, 136 µmol) was added at 0°C to a solution of (*1H*-benzo[d][1,2,3]triazol-5-yl)((*3aR,6aR*)-hexahydropyrrolo[3,4-c]pyrrol-2(*1H*)-yl)methanone hydrochloride (**27**; 40 mg, 136 µmol), *N*-methylmorpholine (82.6 mg, 89.9 µl, 817 µmol), and 3-(4-(trifluoromethoxy)phenyl)propanoic acid (31.9 mg, 136 µmol) in *N,N*-dimethylformamide (5 mL). After 30 min, the ice bath was removed and then after 16 h, the reaction mixture was partitioned between dichloromethane and sat. aq. sodium hydrogen carbonate solution. The organic layer was washed with brine, dried over magnesium sulfate, filtered, and evaporated. Chromatography (silica gel; gradient dichloromethane to dichloromethane / methanol / 25% aq. ammonia solution 90:10:0.25) produced the title compound **14** (57 mg, 88%) as a light-yellow solid.

^1^H NMR (CDCl_3_, 300 MHz) δ 14.24 (br s, 1H), 8.03 (br s, 1H), 7.83 (br d, 1H, J=8.1 Hz), 7.59 (br d, 1H, J=8.7 Hz), 7.2-7.3 (m, 2H), 7.1-7.2 (m, 2H), 3.8-4.1 (m, 2H), 3.5-3.7 (m, 2H), 3.3-3.5 (m, 2H), 2.9-3.2 (m, 4H), 2.5-2.7 (m, 2H), 2.1-2.5 (m, 2H).

LC-HRMS (m/z): [M+H]^+^ calcd for [C_23_H_22_F_3_N_5_O_3_+H]^+^: 474.1748, found: 474.1752, UV purity (230-300 nm): 97%.

S1.13: (*3aS,6aS*)-5-(*1H*-Benzotriazole-5-carbonyl)-hexahydro-pyrrolo[3,4-c]pyrrole-2-carboxylic acid 3-chloro-5-methanesulfonyl-benzyl ester (**15**)

To a solution of (3-chloro-5-(methylsulfonyl)phenyl)methanol (CAS-RN 1513888-30-6; 21.4 mg, 96.9 µmol) in acetonitrile (4.4 mL) was added *N,N'*-carbonyldiimidazole (16.5 mg, 102 µmol,). Then after 3 h, triethylamine (49 mg, 485 µmol) and (*1H*-benzo[d][1,2,3]triazol-5-yl)((*3aR,6aR*)-hexahydropyrrolo[3,4-c]pyrrol-2(*1H*)-yl)methanone hydrochloride (**27**; 32 mg, 97 µmol) were added and the reaction mixture was heated at reflux for 16 h. After cooling, the reaction mixture was partitioned between ethyl acetate and sat. aq. ammonium chloride solution. The organic layer was washed with brine, dried over magnesium sulfate, filtered, and evaporated. Chromatography (silica gel; gradient dichloromethane to dichloromethane/methanol/25% aq. ammonia solution 90:10:0.25) afforded the title compound **15** (35 mg, 72%) as a white foam.

^1^H NMR (CDCl_3_, 300 MHz) δ 12.07 (br s, 1H), 8.05 (s, 1H), 7.8-7.9 (m, 3H), 7.62 (m, 2H), 5.20 (s, 2H), 3.9-4.1 (m, 1H), 3.8-3.9 (m, 1H), 3.5-3.8 (m, 2H), 3.3-3.5 (m, 2H), 3.0-3.2 (m, 5H), 2.2-2.6 (m, 2H).

LC-HRMS (m/z): [M+H]^+^ calcd for [C_22_H_22_ClN_5_O_5_S+H]^+^: 504.1103, found: 504.1107, UV purity (230-300 nm): 100%.

## Data and procedures for X-ray structures of 1, 10 and 13

### Crystal structures of ATX

As rat and human ATX share identical ligand binding and active sites, but rat ATX crystallized more readily in our hands, the rat homologue was used for structural studies. The cDNA coding for residues 1-862 of rat ATX (double variant N53A, N410A to reduce glycosylation) was cloned into pcDNA3.1(‑) neo, encoding a C-terminal His-tag fusion protein. After production in HEK 293-F cells (Thermo Fisher, Waltham, MA, USA) the protein was purified via IMAC on a HisTRAP (Sigma-Aldrich, St. Louis, MO, USA), followed by gel permeation chromatography on a Superdex 200 XK (Sigma-Aldrich) column in 20 mM BICINE/NaOH pH 8.5, 150 mM NaCl, 0.02% NaN_3_. Crystals were grown by sitting drop vapor diffusion in 96-well plates at 298 K. 10-15 mg/mL rat ATX in the above buffer were mixed with reservoir consisting of 11-17% PEG3350, 0.1 M NaOAc pH 4.5, 0.2 M Ca(OAc)_2_, including seeds from older crystal preparations. The ratios of protein and reservoir tested were 50:50 and 70:30 in a total drop volume of 200 nL. Crystals grew to a final size of ca. 0.5 mm within three months and were suitable for soaking with ligands. 0.2 mg of ligand powder was suspended in 5 µL of mother liquor and 0.2 µL of the slurry were applied to a drop with crystals, thus ensuring maximum ligand concentration under the crystallization conditions and avoiding osmotic shock to the crystals. After overnight incubation, crystals were harvested, cryo-protected with paraffin oil, and flash-cooled in liquid nitrogen for data collection at 100 K at Swiss Light Source beamline X10SA (Paul Scherrer Institute (PSI), Villigen, Switzerland), using a wavelength of 1 Å and an oscillation range of 0.1°. Data from the pixel detector were integrated with XDS (Kabsch, 2010), scaled with AIMLESS (Winn et al., 2011), and treated for possible anisotropy with STARANISO (Global Phasing Ltd., Cambridge, UK). Diffraction data were phased by molecular replacement using an in-house ATX starting model. Models were built in COOT (Emsley et al., 2010) and refined with REFMAC5 (Winn et al., 2011). Ligand restraints were generated with GRADE from the BUSTER suite (Blanc et al., 2004), taking statistics from the CSD into account. Crystallographic and model statistics are collected in the Supplementary Table 1. Coordinates and structure factors have been deposited with the PDB under accession codes 5S9L (**1**), 5S9M (**10**), and 5S9N (**13**).

**
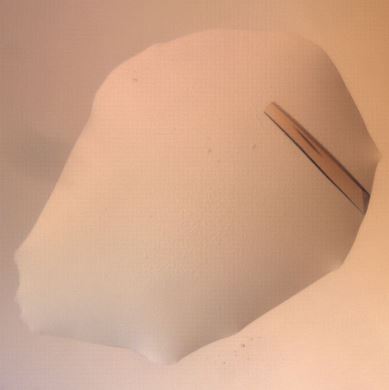
**Typical rat ATX crystal of ca. 0.5 mm length ready for soaking.

## Determination of Lysophosphatidic acids (LPAs) in rat plasma by HPLC-MS/MS

### Introduction and basis for method development

Native levels of phosphatidic acids in plasma are very low (two-digit nM level), while the precursor LPC is highly concentrated. As soon as plasma or blood was kept at elevated temperature, LPA was produced from LPC at a rate corresponding to the autotaxin activity. Another source of artefact LPA formation is a very low pH during extraction (reported for HCl (Baker et al., 2001)). In our hands, acetic acid and formic acid did not increase the LPA content.

If observed, nonlinear calibrations are a consequence of adsorption to surfaces if the content of organic solvents is too low. Therefore, transfers have to be avoided wherever possible and by using the method described below, linear calibrations between 20 and 5000 ng/mL were obtained.

This method was adopted from Scherer et al. (Scherer et al., 2009). It used hydrophilic interaction (HILIC) HPLC and negative ion electrospray detection. Adaptations to the gradient were made and other precautions (intermediate blank injections, special rinsing solvents) were taken to minimize carryover, a common problem in HILIC, to prevent premature clogging of the column and to ensure separation of the LPA peak from the corresponding LPC peak, since in-source fragmentation of a high LPC signal can produce artificial LPA signals.

Since the most influential variable on accuracy and precision in LC/MS are matrix effects, using sample identical material for the preparation of the calibrators (and not e.g. stripped or surrogate material) is crucial for biomarker measurements. In this case the calibrators contain a certain amount of analyte which leads to an y-axis intercept of the calibration line and incorrect results for the unknown values. Data analysis for this method is described below in section 1.3.5.

The calibration samples were prepared by spiking of cold-sampled low-level blank plasma of the study species to levels of 0, 20-5000 ng/mL.

The method was continuously optimized and therefore not systematically validated. In each sequence, one series of calibration samples was analyzed with the unknown samples and injected twice to ensure and check reproducibility.

### Reference Materials

LPA: 16:0, 17:0 (internal standard), 18:1, and 20:4 lysophosphatidic acids were obtained from Avanti polar lipids (Birmingham, AL, USA). Stock and spiking solutions were stored at -20°C. To ensure dissolution they were heated to room temperature and were sonicated for 10 min before use.

Plasma for calibrators: Commercial sample sources were considered inappropriate because of the very high basal LPA values that are produced during conventional sampling. Therefore, blank plasma was obtained from the animal husbandry or the Medical Service for human plasma of F. Hoffmann-La Roche Ltd. (Basel, Switzerland). It was collected into pre-cooled containers, kept on ice, and immediately centrifuged at 4°C. 1-2 mL aliquots were frozen on dry ice and stored at -20°C.

### Sample processing

Samples were kept at -70°C until analysis. 25 µL of rat, human, or mouse plasma were transferred to amber 2 mL glass vials. 25 µL of internal standard solution (5 µM LPA 17:0 in methanol/water 1:1, (or methanol/water 1:1 for the double blank)) were added. 100 µL of methanol were added, vials were capped and mixed on a Thermomixer (Eppendorf SE, Hamburg, Germany) at 4°C for 30 min and centrifuged for 10 min at 4°C and 5000 rpm (ca. 3500 x g). 100 µL of the supernatant were transferred to tapered autosampler vials, crimped, and 10 µL were injected to the LC-MS/MS instrument. The whole sequence was injected twice.

### LC-MS/MS conditions

A Micromass Quattro Ultima II equipped with a UPLC system and a CTC autosampler (all Waters GmbH, Eschborn, Germany) with a cooled sample stack was used. Nitrogen was used for all source gas supplies and Argon as collision gas. The LC column was an Atlantis HILIC Silica of 2.1 x 50 mm dimension and 3 µm particle size (Waters). Phase A was 50 mM ammonium formate with 0.2% formic acid, phase B was acetonitrile with 0.2% of formic acid. The gradient comprised a highly aqueous rinsing period which improved column life, and an extended equilibration period of 6 min: 500 µL flow, 0 min 5%A - 0.7 min 5%A - 1.5 min 25%A - 1.71 min 95%A - 2.0 min 95%A - 2.01 min 5%A – 8 min 5%A. The injector wash was 1) acetonitrile-water 95:5 and 2) methanol-water 1:1. After highly concentrated calibrators, solvent blanks were inserted to minimize carryover effects.

The mass spectrometer was operated in negative electrospray mode, tuned to unit resolution and gas flows and source temperatures were optimized for 500 µL of flow. The HPLC eluent was diverted to waste except during peak elution. In negative ion mode, the glycerophosphate fragment with m/z 153 is by far the most prominent fragment. The following mass pairs were chosen for the analysis:

| **Compound** | **Mass transition (m/z)** | **Cone voltage (V)** | **Collision energy (V)** |
| --- | --- | --- | --- |
| LPA 16:0 | 409.2 - 153.1 | 70 | 25 |
| LPA 17:0 | 423.2 - 153.1 | 80 | 23 |
| LPA 18:0 | 437.2 - 153.1 | 80 | 24 |
| LPA 18:1 | 435.2 - 153.1 | 70 | 25 |
| LPA 20:4 | 457.2 - 153.1 | 70 | 25 |

### Data analysis

A 1/y weighted linear regression was applied to establish the calibration function. At this stage, slope and intercept are correct, but unknown values as well as %accuracies are displayed incorrectly since the analyte content of the blank matrix is not yet considered. To calculate the unknown concentrations, additional processing is required. Therefore, the slope of the calibration line was taken from the instrument software calibration. Then, the peak area ratios were exported and were divided by the slope, which provided the desired absolute sample concentrations for the LPAs. Calculations were done in MS Excel (Microsoft Corporation, Redmond, WA, USA) and graphics in TIBCO Spotfire (TIBCO Software Inc., Palo Alto, CA, USA).

# Supplementary Tables

**Supplementary Table 1: Crystallographic parameters for X-ray structures of 1, 10, and 13.** Values in parentheses are for the highest resolution shell.

|  | **PDB 5S9M**  **Compound 10** | **PDB 5S9N**  **Compound 13** | **PDB 5S9L**  **Compound 1** |
| --- | --- | --- | --- |
| Wavelength (Å) | 0.9999 | 1 | 0.9998 |
| Resolution range | 48.37-1.8 (1.864-1.8) | 45.87-1.8 (1.864-1.8) | 61.62-1.9 (1.968-1.9) |
| Space group | P 21 21 21 | P 21 21 21 | P 21 21 21 |
| Unit cell (a, b, c) (Å) | 83.0, 91.0, 119.0 | 84.1, 91.7, 120.2 | 83.5, 91.3, 118.4 |
| # Total reflections | 555099 (50779) | 574827 (56011) | 508407 (32806) |
| # Unique reflections | 84071 (8306) | 86689 (8565) | 71470 (6671) |
| Multiplicity | 6.6 (6.1) | 6.6 (6.5) | 7.1 (4.9) |
| Completeness (%) | 99.90 (99.89) | 99.92 (99.86) | 99.36 (94.42) |
| Mean I / (I) | 10.50 (1.20) | 11.21 (1.24) | 21.74 (3.59) |
| Wilson B-value | 25.73 | 27.51 | 25.96 |
| R_merge_ | 0.1103 (1.433) | 0.09747 (1.485) | 0.05286 (0.3545) |
| R_meas_ | 0.1198 (1.567) | 0.1058 (1.614) | 0.05693 (0.3966) |
| R_pim_ | 0.04629 (0.6254) | 0.04075 (0.6265) | 0.02085 (0.1736) |
| CC_1/2_ | 0.998 (0.427) | 0.998 (0.578) | 0.999 (0.902) |
| CC* | 1 (0.774) | 1 (0.856) | 1 (0.974) |
| # Reflections for refinement | 84071 (8302) | 86689 (8553) | 71470 (6671) |
| # Reflections for R_free_ | 4273 (450) | 4407 (449) | 3604 (308) |
| R_work_ | 0.1714 (0.2995) | 0.1906 (0.3247) | 0.1581 (0.2042) |
| R_free_ | 0.2107 (0.3218) | 0.2329 (0.3440) | 0.1910 (0.2211) |
| CC_work_ | 0.967 (0.743) | 0.962 (0.788) | 0.965 (0.910) |
| CC_free_ | 0.955 (0.753) | 0.958 (0.807) | 0.957 (0.900) |
| # of non-hydrogen atoms | 7254 | 7093 | 7224 |
| macromolecules | 6573 | 6528 | 6571 |
| ligands | 180 | 136 | 143 |
| solvent | 501 | 429 | 510 |
| # Protein residues | 818 | 816 | 805 |
| RMS bonds (Å) | 0.016 | 0.018 | 0.014 |
| RMS angles (°) | 1.79 | 1.96 | 1.85 |
| Ramachandran favored (%) | 96.27 | 96.51 | 97 |
| Ramachandran allowed (%) | 3.11 | 2.99 | 3 |
| Ramachandran outliers (%) | 0.62 | 0.5 | 0 |
| Rotamer outliers (%) | 3.11 | 3.83 | 0.54 |
| Clashscore | 3.02 | 4.15 | 2.05 |
| Average B-values (Å^2^) | 31.65 | 33.72 | 32.88 |
| macromolecules | 31.01 | 33.33 | 32.52 |
| ligands | 45.58 | 51.67 | 44.17 |
| solvent | 34.97 | 34 | 34.32 |

**Supplementary Table 2: Off-target activity screening for compound 13.** The assays given in the table below were conducted at Eurofins CEREP SA (Celle-Lévescault, France) and were run as single point measurements in duplicates in the presence of 10 µM of compound **13**.

| **Assay (species) [readout]** | **Response at 10 uM of cmpd 13** |  | **Assay (species) [readout]** | **Response at 10 uM of cmpd 13** |  | **Assay (species) [readout]** | **Response at 10 uM of cmpd 13** |
| --- | --- | --- | --- | --- | --- | --- | --- |
| 5HT1A (h)  [% inhib] | -20 |  | HDAC11 (h)  [% inhib] | 0 |  | PCP receptor (r)  [% inhib] | -16 |
| 5HT2a (h)  [% inhib] | 39 |  | HDAC3 (h)  [% inhib] | 1 |  | PDE-5 (h)  [% inhib] | 19 |
| 5HT2B (h)  [% inhib] | -3 |  | HDAC4 (h)  [% inhib] | 3 |  | PPARγ (h), antago. mode  [% inhib] | 6 |
| 5HT3 (h)  [% inhib] | -2 |  | HDAC6 (h)  [% inhib] | -14 |  | PPARγ (h), agonist mode  [% pos ctrl] | 0 |
| Acetylcholinesterase (h)  [% inhib] | -23 |  | Histamine H1 receptor (h)  [% inhib] | 5 |  | Prostaglandin F receptor (h)  [% inhib] | 26 |
| ADAM 17 (h)  [% inhib] | 9 |  | Histamine H2 receptor (h)  [% inhib] | 12 |  | S1P1 (h), antago. mode  [% inhib] | 8 |
| Adenosine A1 respetor (h)  [% inhib] | 1 |  | Histamine H3 receptor (h)  [% inhib] | -10 |  | S1P1 (h), agonist mode [% pos ctrl] | 5 |
| Adenosine A3 respetor (h)  [% inhib] | 33 |  | HIV-1 protease (h)  [% inhib] | 0 |  | S1P2 (h), antago. mode  [% inhib] | 45 |
| Androgen receptor (h)  [% inhib] | -1 |  | LPA1 receptor (h)  [% inhib] | -19 |  | S1P2 (h), agonist mode  [% pos ctrl] | -2 |
| ACE (h)  [% inhib] | -25 |  | LPA2 receptor (h), antago. mode  [% inhib] | 49 |  | S1P3 (h), antago. mode  [% inhib] | 47 |
| Angiotensin II receptor 1 (h)  [% inhib] | 14 |  | LPA2 receptor (h), agonist mode  [% pos ctrl] | 8 |  | S1P3 (h), agonist mode  [% pos ctrl] | 8 |
| Ca2^+^ channel (diltiazem site) (r)  [% inhib] | 9 |  | LPA3 receptor (h), antago. mode  [% inhib] | 56 |  | Serotonin transporter (h)  [% inhib] | 28 |
| Carbonic anhdrase II (h)  [% inhib] | -6 |  | LPA3 receptor (h), agonist mode [% pos ctrl] | -3 |  | Sigma (h)  [% inhib] | 38 |
| CDK2 (h)  [% inhib] | 0 |  | ML3R (h)  [% inhib] | 48 |  | Sodium channel (site 2) (r)  [% inhib] | 49 |
| Cyclooxygenase 2 (h)  [% inhib] | 1 |  | MMP-1 (h)  [% inhib] | -9 |  | Somatostatin receptor 4 (h)  [% inhib] | 12 |
| Dopamine D1 receptor (h)  [% inhib] | 0 |  | MMP-3 (h)  [% inhib] | 3 |  | Xanthine oxidase (b)  [% inhib] | 7 |
| Dopamine D2 receptor (h)   [% inhib] | 4 |  | MMP-9 (h)  [% inhib] | -22 |  | ZAP70 (h)  [% inhib] | 9 |
| ECE-1 (h)  [% inhib] | 16 |  | MAO-A (h)  [% inhib] | -1 |  | Alpha1A-adrenoreceptor (h)  [% inhib] | 6 |
| Estrogen receptor alpha (h)  [% inhib] | -6 |  | MAO-B (h) [% inhib] | 6 |  | Alpha2A-adrenoreceptor (h)  [% inhib] | 3 |
| GABA-A (benzodiazepine site) (r)  [% inhib] | -26 |  | Muscarinic receptor M2 (h)  [% inhib] | -15 |  | Beta1-adrenoreceptor (h)  [% inhib] | 2 |
| Glucocorticoid receptor (h)  [% inhib] | 6 |  | Muscarininc receptor M4 (h)  [% inhib] | -17 |  | kappa opioid receptor (r)  [% inhib] | -1 |
| Glycine receptor (strychnine insensitive) (r)  [% inhib] | 17 |  | Neprilysin (neutral endopeptidase) (h)  [% inhib] | -29 |  | mu opioid receptor (h)  [% inhib] | 15 |
| GSK-3A (h)  [% inhib] | -11 |  | Nicotinic receptor (muscle) (h)   [% inhib] | 3 |  | PCP receptor (r)  [% inhib] | -16 |
| GSK-3B (h)  [% inhib] | -8 |  | Norepinephrine transporter (h)  [% inhib] | 20 |  | PDE-5 (h)  [% inhib] | 19 |

# Supplementary Figures

**Supplementary Figure 1: IOP in normal ranges in the EAG model.** IOP measurements one week before and 1, 2, and 3 weeks after immunization revealed no alterations throughout the whole study and showed no difference between the groups at all points in time. N=5-6 animals/group. Values are mean±SEM±SD.


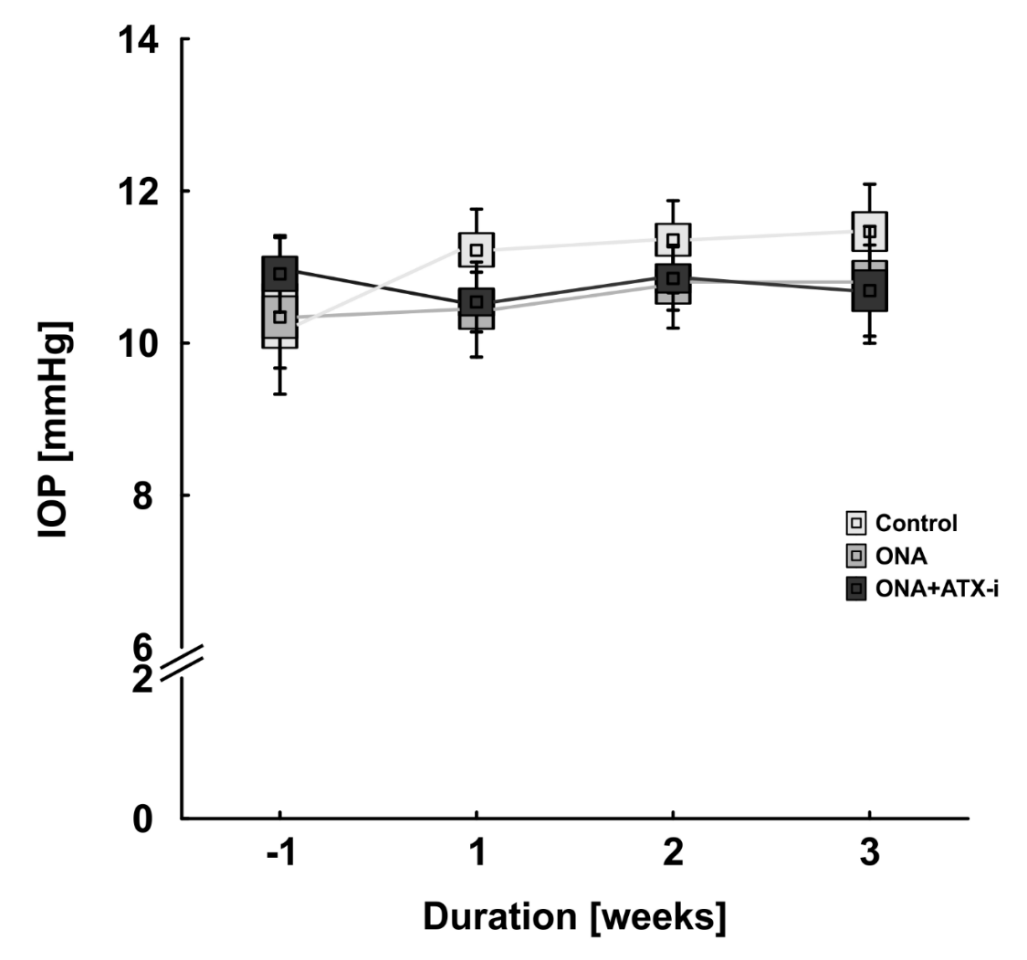


# Supplementary References

Baker, D.L., Desiderio, D.M., Miller, D.D., Tolley, B., and Tigyi, G.J. (2001). Direct Quantitative Analysis of Lysophosphatidic Acid Molecular Species by Stable Isotope Dilution Electrospray Ionization Liquid Chromatography-Mass Spectrometry. *Analytical Biochemistry* 292**,** 287-295.

Blanc, E., Roversi, P., Vonrhein, C., Flensburg, C., Lea, S.M., and Bricogne, G. (2004). Refinement of severely incomplete structures with maximum likelihood in BUSTER-TNT. *Acta Crystallogr D Biol Crystallogr* 60**,** 2210-2221.

Emsley, P., Lohkamp, B., Scott, W.G., and Cowtan, K. (2010). Features and development of Coot. *Acta Crystallogr D Biol Crystallogr* 66**,** 486-501.

Kabsch, W. (2010). Xds. *Acta Crystallogr D Biol Crystallogr* 66**,** 125-132.

Scherer, M., Schmitz, G., and Liebisch, G. (2009). High-Throughput Analysis of Sphingosine 1-Phosphate, Sphinganine 1-Phosphate, and Lysophosphatidic Acid in Plasma Samples by Liquid Chromatography-Tandem Mass Spectrometry. *Clin Chem* 55**,** 1218-1222.

Winn, M.D., Ballard, C.C., Cowtan, K.D., Dodson, E.J., Emsley, P., Evans, P.R., Keegan, R.M., Krissinel, E.B., Leslie, A.G., Mccoy, A., Mcnicholas, S.J., Murshudov, G.N., Pannu, N.S., Potterton, E.A., Powell, H.R., Read, R.J., Vagin, A., and Wilson, K.S. (2011). Overview of the CCP4 suite and current developments. *Acta Crystallogr D Biol Crystallogr* 67**,** 235-242.
